# Effects of the Denitrification Inhibitor “Procyanidins” on the Diversity, Interactions, and Potential Functions of Rhizosphere-Associated Microbiome

**DOI:** 10.3390/microorganisms9071406

**Published:** 2021-06-29

**Authors:** Mylène Hugoni, William Galland, Solène Lecomte, Maxime Bruto, Mohamed Barakat, Florence Piola, Wafa Achouak, Feth el Zahar Haichar

**Affiliations:** 1 VetAgro Sup, UMR Ecologie Microbienne, Université Lyon, Université Claude Bernard Lyon 1, CNRS, INRAE, F-69622 Villeurbanne, France; mylene.hugoni@univ-lyon1.fr (M.H.); galland.william@outlook.fr (W.G.); solenelecomte5@gmail.com (S.L.); 2 Institut Universitaire de France (IUF), CEDEX 05, F-75231 Paris, France; 3 Laboratoire de Biométrie et Biologie Évolutive, Université Lyon, Université Lyon 1, CNRS, UMR5558, 43 bd du 11 Novembre 1918, F-69622 Villeurbanne, France; maxime.bruto@univ-lyon1.fr; 4 Laboratory of Microbial Ecology of the Rhizosphere (LEMiRE), Aix Marseille University, CEA, CNRS, BIAM, F-13108 Saint-Paul-Lez-Durance, France; mohamed.barakat@cea.fr (M.B.); wafa.achouak@cea.fr (W.A.); 5 Université Lyon, Université Claude Bernard Lyon 1, CNRS, ENTPE, UMR 5023 LEHNA, F-69622 Villeurbanne, France; florence.piola@univ-lyon1.fr; 6 Microbiologie, Adaptation, Pathogénie, INSA-Lyon, Université Claude Bernard Lyon 1, CNRS, UMR5240, 10 rue Raphaël Dubois, F-69622 Villeurbanne, France

**Keywords:** denitrification inhibitor, procyanidins, rhizosphere microbiome, diversity, functions, network

## Abstract

Some plant secondary metabolites, such as procyanidins, have been demonstrated to cause biological denitrification inhibition (BDI) of denitrifiers in soils concomitantly with a gain in plant biomass. The present work evaluated whether procyanidins had an impact on the diversity of nontarget microbial communities that are probably involved in soil fertility and ecosystem services. Lettuce plants were grown in two contrasting soils, namely Manziat (a loamy sand soil) and Serail (a sandy clay loam soil) with and without procyanidin amendment. Microbial diversity was assessed using Illumina sequencing of prokaryotic 16S rRNA gene and fungal ITS regions. We used a functional inference to evaluate the putative microbial functions present in both soils and reconstructed the microbial interaction network. The results showed a segregation of soil microbiomes present in Serail and Manziat that were dependent on specific soil edaphic variables. For example, Deltaproteobacteria was related to total nitrogen content in Manziat, while Leotiomycetes and Firmicutes were linked to Ca^2+^ in Serail. Procyanidin amendment did not affect the diversity and putative activity of microbial communities. In contrast, microbial interactions differed according to procyanidin amendment, with the results showing an enrichment of Entotheonellaeota and Mucoromycota in Serail soil and of Dependentiae and Rozellomycetes in Manziat soil.

## 1. Introduction

Today, agriculture is facing a major challenge of feeding more and more people in shorter and shorter production times [1]. This increasingly intensive agriculture uses a large number of nitrogen fertilizers in order to increase yields [2]. These fertilizers are used because nitrogen is one of the most important and limiting factors for plant growth [3,4]. Nitrogen (N) in the form of nitrate causes pollution problems that can affect the environment as well as human health [5,6]. Indeed, the nitrate used for fertilization is not totally absorbed by the crops due to limited plant metabolism or excess application [7]. As a result, there is an increased risk of nitrate leaching into the soil [8], which can lead to 20% loss of N in the system [9]. This phenomenon can lead to an overconcentration of this element in water tables, water reservoirs, streams, lakes, and rivers [10], thereby causing the eutrophication phenomenon as a consequence of the over-abundance of nutrients for macroscopic plants, microalgae, and bacteria consuming a lot of oxygen [11]. In the long term, these imbalances have harmful results, such as development of undesirable or toxic algae or bacteria, asphyxiation of fish, and reduction of the specific richness of the environment and therefore the biodiversity [12,13]. In addition, nitrate in soils is also consumed by so-called denitrifying microorganisms via the denitrification process [14]. This can account for up to 60% of the N loss in farming systems [15]. During this process, nitrate is transformed into N_2_O released from soil and water. N_2_O is known to be a powerful greenhouse gas that is 1000 times less concentrated than CO_2_ but with up to 300 times greater global warming potential [16]. It contributes to nearly 7.5% of the increase in the greenhouse effect [17], with its main source being microbial denitrification activity [18]. Moreover, it is recognized that water that is overconcentrated in nitrate as well as some cultures known to accumulate more nitrate in their tissues [19,20] can eventually cause health problems for consumers if the doses are too high [21,22]. In order to reduce the risk to the environment and human health, modern agriculture is increasingly exploring the use of other products, such as biostimulants or inhibitors, in order to limit N input while maintaining a viable productivity rate [23,24].

One of the solutions would be to act on soil microorganisms related to the N cycle in order to limit the loss of N from agrosystems through the release of greenhouse gases or leaching. Several methods exist and have been tested, including the use of 3,4-dimethylpyrazole phosphate (DMPP) and dicyandiamide (DCD), which are molecules capable of blocking or delaying nitrification [25,26]. These molecules can thus prevent the conversion of ammonium from soil into nitrate, which is more easily assimilated by microorganisms or subject to leaching losses, thereby increasing the efficiency of ammonium-based fertilizers for crop productivity [27]. These nitrification inhibitors have been shown to reduce N_2_O emissions and nitrate leaching in the field across different zones and climates [28,29,30]. However, compounds such as DMPP deeply affect the microbial communities by limiting their growth and their capacity to perform different ecological processes and to utilize a wide assortment of substrates [31].

In addition to these chemical molecules acting on nitrification, it is also possible to act directly on the denitrification process. Indeed, it has been shown that some plants have the capacity to exude a flavonoid called procyanidins at the root level, which is able to inhibit soil denitrifying bacteria [32]. This phenomenon, known as biological denitrification inhibition (BDI), limits the competition between denitrifying bacteria and plants for nitrate. This increases the pool of this nitrogenous compound available in the soil, thus proving beneficial for plants [33]. In addition, it has been shown that amendment of agricultural soil cultivated with lettuce and celery induced better growth yield of these plants due to BDI and the subsequent increase in the nitrate available in soil for plant growth [34]. A significant decrease in the abundance of denitrifiers was observed in plots amended with procyanidins, suggesting a decrease in competitiveness or a counter selection of denitrifiers following BDI [35]. Recently, Galland et al. (2021) demonstrated that in two different type of soils (loamy sand and sandy clay loam soils) cultivated with lettuce in the field, the addition of procyanidins caused inhibition of denitrification, an increase in available nitrate, counterselection of denitrification communities, and a gain in plant mass without modification of the soil structure [36]. Hence, the use of procyanidins in the field represents a more environmentally friendly and sustainable agricultural alternative by limiting the use of fertilizers and reducing N losses in the soil while increasing plant growth and productivity. For the prospect of a more sustainable method that does not affect soil fertility, it will be interesting to examine whether procyanidin amendment impacts soil microbiome involved in its fertility and thus participates in ecosystem services.

In this study, we aimed to test the effect of procyanidin amendment on lettuce-associated microbiome cultivated in contrasting soils, namely a sandy clay loam soil texture from the Serail Experimental Station and a loamy sand texture from the agricultural field in Manziat. To achieve our purpose, the same variety of lettuce was planted during summer 2018 concomitantly in both Serail and Manziat that were amended or not with procyanidins at two concentrations (0 and 210 kg ha^−1^). The bacterial and fungal communities were monitored by Illumina sequencing using 16S rRNA gene and ITS regions, respectively. Functional inference of microbial abilities was predicted, and microbial networks were analyzed.

## 2. Materials and Methods

### 2.1. Plant Growth, Experimental Design, and Harvesting

**Figure 1:**
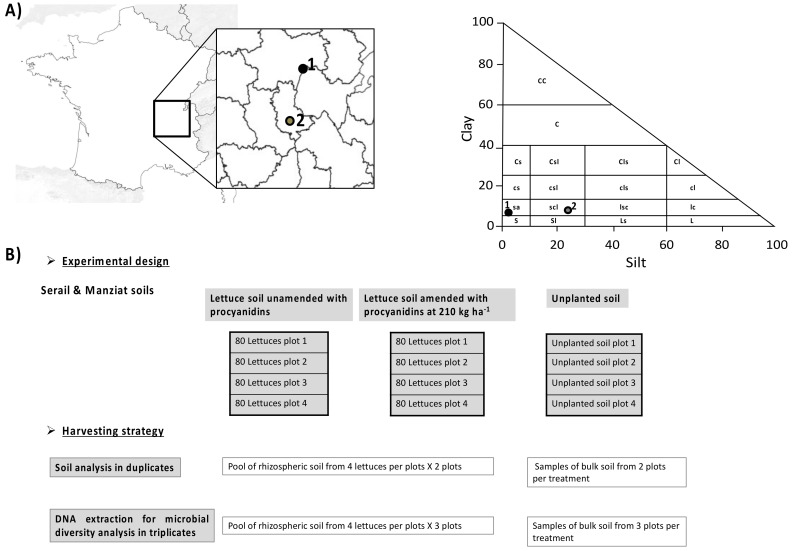
(**A**) Location of experimental sites and distribution of soil texture according to Hénin (1976) [38]. The number 1 indicates Manziat soil and 2 indicates Serail soil. S: coarse sand; s: fine sand; L: coarse silt; l: fine silt; C: coarse clay; c: fine clay. (**B**) Experimental design and harvesting strategy.

Lettuce (*Lactuca sativa* var Loubresac, Earl LIMOUSIN) was grown in two experimental fields following a Fisher system [37] with 4 plots per treatment. The fields were located in agricultural land in Manziat (01570 Ain, France) (46°22’2.28″ N, 4°54’100 3.671″ E) with loamy sand soil (sand: 82.5%, silt: 8.9%, and clay: 8.5%) and at the Serail Experimental Station (Brindas, 69126 Rhône, France) (45°43’46.4″ N, 4°43’37.1″ E) with a sandy clay loam soil (sand: 68.7%, silt: 21.9%, and clay: 9.5%) (Figure 1A). The type of soil was determined according to [38]. The experimental design (Figure 1B) was the same in both fields, with 4 plots for each treatment, namely unamended planted soil with lettuce, amended planted soil with lettuce, and unplanted soil (bulk soil). Lettuce was planted in 4 parallel rows of 20 in 6 m × 1.4 m plots (8.4 m^2^) (planted soil with lettuce), with an unplanted area of 2 m × 1.4 m (2.8 m^2^) corresponding to unplanted soil (bulk soil). For planted soil amended with procyanidins, a concentration of about 210 kg ha^−1^ was used as the most effective concentration as determined in a previous study by Galland et al. (2019). Both sites were watered with 3 mm of water each day during the first week and with 8 mm of water per day for the next 10 days. Then, until the end of the experiment, 12 mm of water was applied on the fields every two days. Procyanidins were added 2 weeks after planting (stage with 7–9 leaves) to soil whose nitrate had been brought to the ZENIT grid, i.e., 40 kg NO_3_^−^ ha^−1^ [39]. Commercial procyanidins (Laffort TANIN VR GRAPE^®^, Bordeaux, France) were applied in aqueous solution (standard water) by 2 nozzle spray booms to provide 500 L ha^−1^ or 0.42 L per plot between the lettuce rows, and the soil was then hoed. Each site was binned and watered (with 8 mm) just after the addition of procyanidins.

At the end of plant growth, the roots of each lettuce, retrieved from amended and unamended plots, were separated manually from the root-adhering soil (RAS), which was collected and frozen in liquid N_2_ immediately and stored at −80 °C. Samples from unplanted soil plots were also collected and stored at −80 °C.

### 2.2. Measurement of Environmental Variables

For the soil analysis (Figure 1B), 3 treatments per site (planted soil with and without procyanidins and unplanted soil) in duplicates were considered with each replicate corresponding to a pool of rhizospheric soil retrieved from 2 plots. Physical and chemical soil characteristics were performed by CESAR (Centre Scientifique Agricole Regional, Ceyzeriat, 01250 Ain, France) using a standard method (AFNOR, 2005).

### 2.3. DNA Extraction

Total DNA was extracted from 0.5 g of root-adhering soil (RAS) from 3 treatments (planted soil with and without procyanidin amendment and unplanted soil) in triplicates (Figure 1B) using a Fast DNA Spin Kit for Soil (MP Biomedical, Solon, OH, USA). The amount of DNA extracted was then estimated using a Quant-iT PicoGreen^®^ dsDNA Assay kit (Molecular Probes, Carlsbad, CA, USA). Additionally, a DNA extraction was carried out without any biological matrix and considered as a negative control to evaluate ambient contaminations.

### 2.4. Amplicon Libraries and Illumina Sequencing

Two sets of primers were used to analyze either the prokaryotic or the fungal communities retrieved in lettuce.

For prokaryotic community analyses, including both archaea and bacteria, amplification of the V3–V5 region of 16S rRNA genes was performed using the universal primers 515F (5′- α GTG-YCA-GCM-GCC-GCG-GTA -3′ [40] and 909R (5′- β CCC-CGY-CAA-TTC-MTT-TRA-GT -3’ [41]). The PCR mix consisted of 0.5 U of Taq DNA polymerase (Invitrogen), 1X PCR buffer, 1.5 mM of MgCl_2_, 0.8 μM of each primer, 0.2 mM of each dNTP, 8 μg of BSA (New England Biolabs), and 20 ng of genomic DNA in a final volume of 30 μL. All amplifications were carried out in triplicates on a Biorad C1000 thermal cycler (Biorad) using a PCR program composed of 10 min at 94 °C, 35 cycles of 1 min at 94 °C, 1 min at 58 °C, and 1 min 30 s at 72 °C, followed by 10 min at 72 °C.

Regarding the fungal community analyses, the ITS-2 region was specifically amplified using the fITS7 (5′- α GTG ART CAT CGA ATC TTT G -3′) and ITS4 (5′- β TCC TCC GCT TAT TGA TAT GC -3′) primers [42,43]. Indeed, among all regions of the ribosomal cistron, the internal transcribed spacer (ITS) region has the highest probability of successful identification for the broadest range of fungi [44]. All PCR amplifications were carried out in triplicate in a 25 µL reaction mix containing 1 μL of DNA template, 1X PCR buffer (Invitrogen, Illkirch, France), 1.5 mM of MgCl_2_ (Invitrogen), 0.2 mM of each dNTP (ThermoFisher Scientific, Illkirch, France), 0.8 µM of each primer (Life Technologies, Saint-Aubin, France), 0.2 mg.mL^−1^ of bovine serum albumin (New England Biolabs, Evry, France), and 0.5 U of Taq DNA polymerase (Invitrogen). All amplifications were carried out on a Biorad C1000 thermal cycler (Biorad, Nanterre, France) with one cycle of 3 min at 94 °C, followed by 35 cycles of 45 s at 94 °C, 45 s at 55 °C, 45 s at 72 °C, and a final extension step of 7 min at 72 °C.

For both primer sets, α and β represent the two Illumina overhanging adapter sequences (TCG TCG GCA GCG TCA GAT GTG TAT AAG AGA CAG and GTC TCG TGG GCT CGG AGA TGT GTA TAA GAG ACA G for α and β, respectively) allowing the construction of amplicon libraries by a two-step PCR. The three PCR replicates from each sample were pooled, purified with Agencourt AMPure XP PCR Purification kit (Beckman Coulter, Villepinte, France), and quantified using the Quant-iT Picogreen dsDNA Assay kit (Life Technologies, New York, NY, USA). Sequencing on the Illumina MiSeq platform (2 × 300 bp paired-end reads) was performed by Biofidal (Vaulx-en-Velin, France; http://www.biofidal-lab.com) (accessed on 27 June 2021).

### 2.5. Bioinformatic Analysis

Prokaryotic 16S rRNA gene and fungal ITS paired-end reads were merged with a maximum of 10% mismatches in the overlap region using Vsearch [45]. Denoising procedures consisted of discarding reads with no expected length (i.e., expected size between 250 and 580 bp for 16S rRNA genes and between 200 and 600 bp for fungal ITS) and the ones containing ambiguous bases (N). After dereplication, a clusterization tool that runs with SWARM [46] using a local clustering threshold, and not a global clustering threshold as in other software, was employed. In the present work, the aggregation distance equaled 3. Chimeras were then removed using Vsearch [45], and low abundance sequences were filtered at 0.005% (i.e., keeping OTUs with at least 0.005% of all sequences) [47] to discard singletons from the datasets. Taxonomic affiliation was performed with both RDP classifier [48] and Blastn+ [49] against the SILVA database v.132 [50] for the prokaryotic 16S rRNA genes and against the UNITE database v.8.0 [51] for the fungal ITS. This procedure was automated in the FROGS pipeline [52]. All OTUs retrieved in the control condition sample were discarded from the datasets. Then, a normalization procedure was applied to compare samples. The prokaryotic dataset was randomly resampled down to 7541 sequences and the fungal dataset down to 32,983 sequences.

### 2.6. Statistical Analyses

For each dataset (prokaryotes and fungi), we tested the relative importance of different sources of variability (soil, treatment, and presence of lettuce) on the composition of microbial communities using nonparametric permutation-based multivariate analysis of variance (PERMANOVA, function adonis in R package vegan [53]) with abundance-based Bray–Curtis dissimilarity matrices. A stress value was calculated to measure the difference between the ranks on the ordination configuration and the ranks in the original similarity matrix for each repetition [54]. An acceptable stress value should be below 0.1. We also created an ordination of the samples using an NMDS approach with Bray–Curtis dissimilarity matrices (function metaMDS in R package vegan). Distribution of the most abundant prokaryotic orders and fungal genera (>0.04% of total 16S rRNA gene and ITS sequences, respectively) in each condition was evaluated using a heatmap coupled to hierarchical clustering. Common OTUs and specific ones were determined through the construction of Venn diagrams (http://bioinformatics.psb.ugent.be/webtools/Venn/) (accessed on 03 December 2020).

To explain the prokaryotic and fungal community structure for different conditions in the present work, a canonical correspondence analysis (CCA) was performed linking community composition (inferred from prokaryotic phyla and fungal classes representing >1% of sequences in their respective dataset) and a combination of 9 environmental variables (sand, silt, clay, organic carbon, organic matter, total nitrogen, C/N ratio, calcium, and cation exchange capacity (CEC)). This analysis was performed with the vegan package (http://cran.r-project.org/web/packages/vegan/index.html) (accessed on 03 December 2020) in R software.

### 2.7. Functional Inference

The functional potential of the prokaryotic community was predicted with R package Tax4Fun based on taxonomy and abundancy of 16S rRNA OTUs (Aßhauer et al., 2015) using a probabilistic model to assign multiple reference genomes in the SILVA database (v.123). For the subsequent transformation of SILVA-based genera to prokaryotic KEGG organisms, the latest available association matrix based on SILVA release 123 was used according to the programmer’s instructions. Taxonomic abundances were transformed to metabolic capabilities using the precomputed Kyoto Encyclopedia of Genes and Genomes (KEGG) pathway reference profiles according to the MoP approach [55]. A linear discriminant analysis (LDA) effect size (LEfSe) was used [56] to identify the obtained categories that statistically differed according to the soil (Manziat versus Serail) considering lettuce planted with or without procyanidins as well as the obtained categories that varied according to procyanidin treatment.

The tool FUNGuild was used to taxonomically parse fungal OTUs into several ecological categories (i.e., animal pathogens, plants pathogens, wood saprotrophs, etc.) [57] defining eight trophic modes [58]. Only fungal OTUs matching a reference sequence with ≥95% sequence similarity were retained for assignment to guilds using highly probable and possible confidence ranks.

### 2.8. Microbial Networks

Microbiome networks were built as previously described [59] using a pairwise correlation-based approach. Briefly, we computed pairwise correlation coefficients calculated between OTUs using the Pearson method. We permuted the OTU table many times (100 times) to avoid including false positives in the network. We calculated a *p* value for each possible pairwise interaction to test the validity of the detected interaction, with the threshold set at 0.01. After applying the Benjamini–Hochberg correction for multiple tests, the corrected *p* values were converted to an adjacency matrix, from which the microbiome network was constructed. To detect hubs corresponding to nodes with a significantly larger number of connections, we calculated hub centrality scores. The centrality vectors were sorted to select the top 20 OTUs with the highest probability of being keystone species. To explore the global properties of networks, different metrics were estimated, including the number of nodes and edges, diameter, density and transitivity, and average path length. The graphical analysis was performed using the igraph R package.

## 3. Results

### 3.1. Soil Type: Predominant Factor Affecting Microbiome Richness and Diversity

Differences in soil proprieties were found between both unplanted soils with more organic matter and organic carbon in Manziat soil compared to Serail soil. After lettuce growth, organic and carbon matter was enhanced in Serail soil, while it remain unchanged in Manziat soil. Procyanidin amendment had no impact on soil proprieties for Manziat soil but enhanced organic matter concentration in unplanted soil.

To assess the influence of procyanidin amendment on soil microbiota, 16S and ITS metabarcoding was used to reveal the microbial community’s patterns. Rarefaction curves indicated that the sequencing effort was sufficient to describe fungal diversity, while in the prokaryotic dataset, some samples did not appear to have reached an asymptote (Figure S1). To gain insights into the effect of procyanidin amendment on lettuce-associated microbiome, we analyzed the OTU richness (Chao1 index) and diversity (Shannon index) and did not detect significant differences between the different treatments (Table 1).

**Figure 2:**
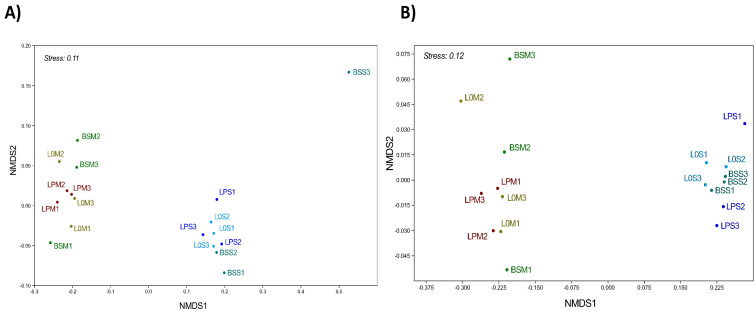
Differences in the structure of prokaryotic (**A**) and fungal (**B**) communities. NMDS ordination was performed with Bray–Curtis dissimilarity matrices. Prokaryotic community structure was evaluated at the OTU level (16S rRNA genes), and fungal community structure was investigated at the OTU level (fungal ITS-2 region). BSM and BSS: bulk soil in Manziat and Serail, respectively. L0M and LPM: lettuce planted in Manziat soil without and with procyanidin amendment, respectively. L0S and LPS: lettuce planted in Serail soil without and with procyanidins, respectively. Numbers 1, 2, and 3 indicate the replicate number.

To evaluate the effects of lettuce seeding, procyanidin amendment, and/or soil type on the taxonomic composition of prokaryotic and fungal communities, we performed a permutational multivariate analysis of variance (PERMANOVA). This analysis was performed on two different levels of either OTU abundance (i.e., number of sequences) or OTU richness (i.e., presence/absence matrix). The same trends were observed when considering the abundance and richness of OTUs (Table 2). The soil type was the predominant factor with a significant effect on prokaryote and fungal abundance and richness, with 45.7 and 54.1% of the variance explained for prokaryotes and fungal OTUs abundance, respectively, and 60.9 and 79.0% of the variance explained for prokaryote and fungal richness, respectively (Table 2, Table S1). This was confirmed using a nonmetric dimensional scaling analysis (NMDS) at the OTU level for prokaryote and fungal abundance datasets (Figure 2).

### 3.2. Microbiome Composition Among Treatments

**Figure 3:**
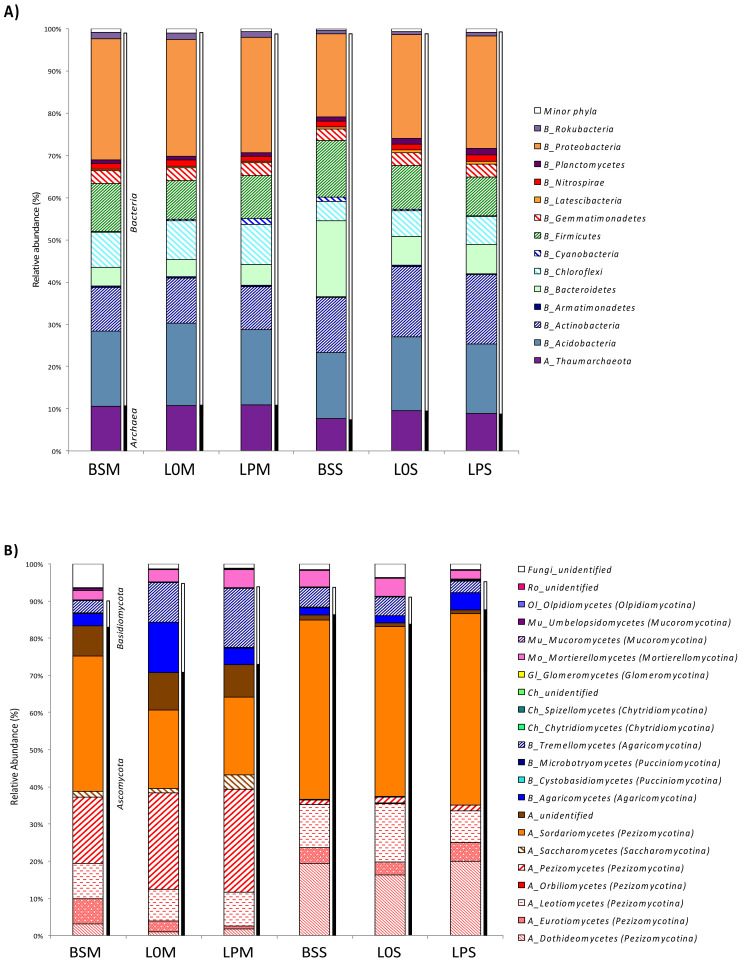
Prokaryotic (**A**) and fungal (**B**) taxonomic composition. Relative abundance (% of sequences) among prokaryotic 16S rRNA gene (at the phylum level) and fungal (at the subphylum/class level) datasets are presented. Minor phyla consisted of very rare bacteria and archaea. BSM and BSS: bulk soil in Manziat and Serail, respectively. L0M and LPM: lettuce planted in Manziat soil without and with procyanidin amendment, respectively. L0S and LPS: lettuce planted in Serail soil without and with procyanidins, respectively.

We investigated the impacts of the different treatments performed in the present work on the taxonomic composition of both prokaryote and fungal communities. Among the prokaryote dataset, the proportion of archaea over bacteria varied according to the soil considered, i.e., Manziat versus Serail (Table S1, Figure 3A). Archaea constituted, on average, 10.9% of the total sequences in Manziat, while they represented only 8.7% of the sequences in Serail soil. More precisely, 14 phyla of archaea and bacteria represented 98.95 to 99.64% of the total sequences across the samples, with minor classes grouped among the minor phyla. Proteobacteria were the most abundant phyla. They were slightly enriched in Serail soil, where they accounted for 27.28 to 28.61% of sequences, while they accounted for 19.57 to 26.64% of sequences in Manziat soil (Figure 3A). The same pattern was observed for Acidobacteria, Thaumarchaeota, Actinobacteria, and Chloroflexi, which accounted for, on average, 18.34, 10.76, 10.49, and 8.96% of the prokaryotic sequences in Serail soil and 16.56, 8.69, 15.30 and 5.82% of the sequences in Manziat soil, respectively. In contrast, other phyla were more abundant in Manziat soil compared to Serail soil, such as Firmicutes or Bacteroidetes, which accounted for, on average, 11 and 10.55% of sequences in Serail soil and only 10.25 and 4.52% of sequences in Manziat soil (Figure 3A). Noticeably, there were no significant changes in abundance between the different samples (Figure 3A), except that Bacteroidetes were enriched (17.94% of sequences) in Manziat soil without lettuce and without procyanidin amendment. Analysis evaluating the effect of lettuce seeding and procyanidin amendment on fungal community in each soil showed that in Manziat, the lettuce seeding had a significant effect on the composition of fungal communities (based on abundance matrix, see Table S2). Ascomycota largely dominated all samples, accounting for 72.9 to 87.6% of the fungal sequences (Figure 3B). Other fungal phyla were also retrieved, such as Basidiomycota, which represented 20.6 and 24.2% of sequences in Manziat soil seeded with lettuce with and without procyanidin amendment, respectively, while they accounted for only 6.8% of the sequences in the same soil without lettuce and without procyanidin amendment. In Serail soil, Basidiomycota represented 7.0 to 7.8% of the sequences (Figure 3B). The less abundant phyla included Mortierellomycota (averaging 3.8% of fungal sequences), Chytridiomycota, Mucoromycota, Olpidiomycota, Rozellomycota, and unidentified fungi. At the finest taxonomic resolution, a total of 22 different fungal classes were identified in the different samples (Figure 3B). On average, certain classes were more abundant in Manziat soil, such as Pezizomycetes, Tremellomycetes, unidentified Ascomycota, Agaricomycetes, unidentified Fungi, and Saccharomycetes, which represented 23.89, 9.98, 8.92, 7.08, 2.15, and 2.21% of the fungal sequences in Manziat soil, respectively, compared to only 1.41, 4.45, 1.03, 2.91, 2.39, and 0.11 % of the sequences in Serail soil (Figure 3B). On the other hand, other classes were dominant in Serail soil, such as Sordariomycetes, Dothideomycetes, Leotiomycetes, and Eurotiomycetes, which accounted for 48.55, 18.56, 11.99, and 4.26% of the sequences in the Serail dataset, respectively, compared to only 26.13, 1.94, 9.05, and 3.51% of the sequences in Manziat soil (Figure 3B).

**Figure 4:**
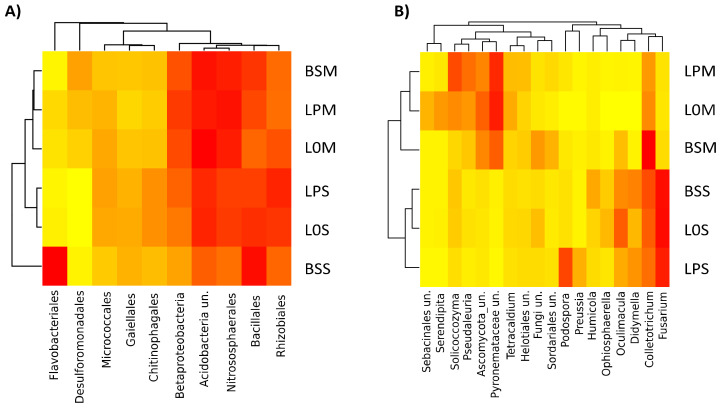
Distribution of the most abundant prokaryotic orders (**A**) and fungal genera (**B**) (>0.04% of total 16S rRNA gene and ITS sequences, respectively) in each condition. The heatmaps are coupled to a hierarchical clustering analysis of the most abundant prokaryotic orders and fungal genera colonizing lettuce procyanidins in Manziat (LPM), lettuce without procyanidins in Manziat (L0M), and bulk soil in Manziat (BSM) as well as lettuce procyanidins in Serail (LPS), lettuce without procyanidins in Serail (L0S), and bulk soil in Serail (BSS).

Using discriminant analysis, we found clear differences in the most abundant fungal genera (>0.04% of total sequence abundance) between Manziat and Serail soil (Figure 4B). Some genera were more specifically associated with one soil type, such as uncultured Pyronemataceae, uncultured Ascomycota, and Solicoccozyma, which were mostly retrieved in Manziat soil, whereas Fusarium, Colletotrichum, and Oculimacula were more specifically associated with Serail soil. A clear shift in fungal abundance was observed between unplanted soil and lettuce planted soil, evidencing a rhizosphere effect. In addition, some genera were found in abundance after procyanidin amendment, such as Podospora in Serail soil and Solicoccozyma in Manziat soil. Other genera were less abundant after procyanidin amendment, such as uncultured Sebacinales and Serendipita in Serail soil. Even if some differences were observed between Serail and Manziat soils for prokaryotes, differences were less clear than for fungi (Figure 4A). Interestingly, the order of Flavobacteriales was more specifically associated with Serail soil (14%) than with Manziat soil (0.3%). Bacillales were more enriched in Serail soil (13%) than in Manziat soil (9.5%). Overall, after procyanidin amendment, the abundance of certain orders was slightly enhanced while other were slightly reduced (Figure 4A). For example, Rhizobiales were enhanced after procyanidin amendment on lettuce planted in Serail soil, while archaeal Nitrososphaerales were reduced after procyanidin amendment on lettuce planted in Serail soil.

### 3.3. Core Microbiome and Differential Microbial Community Composition among Amended and Unamended Treatments

**Figure 5:**
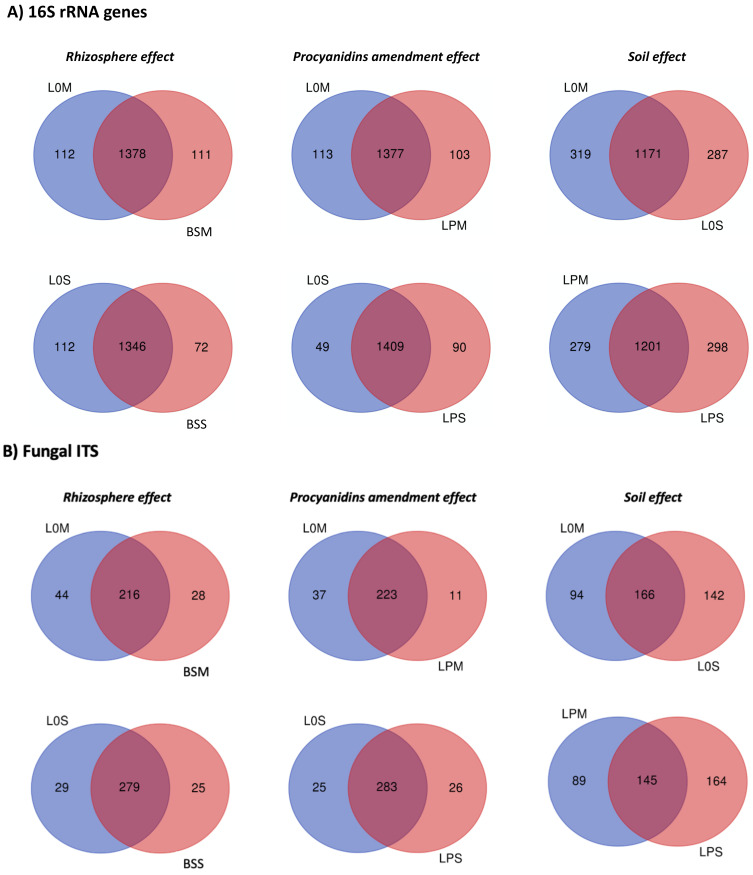
Venn diagrams presenting the shared and specific OTUs detected in 16S rRNA gene (**A**) and fungal (**B**) datasets. For each soil, shared and specific OTUs were determined between lettuce unamended soils (L0) versus bulk soil (BS) to depict the rhizosphere effect and between lettuce unamended soils (L0) and lettuce with procyanidins (LP) to evaluate the effect of procyanidin amendment. Among soils, the values were determined for the lettuce unamended soils (L0) and then for the lettuce with procyanidins (LP) to decipher the soil effect.

To evaluate the existence of common, shared, and specific OTUs among treatments, the distributions of OTUs were analyzed (Figure 5). For prokaryotes (Figure 5A), in Manziat soil, 1378 OTUs were shared between unplanted and planted soil, 112 OTUs were present only om lettuce plant, and 111 OTUs were present only om unplanted soil. After procyanidin amendment, 1377 OTUs were shared by amended and unamended lettuce, whereas only 113 and 103 OTUs were specific to unamended and amended lettuce, respectively. For Serail soil, 1346 OTUs were shared by unplanted and planted soil, 112 OTUs were present only om lettuce plant, and 72 OTUs were present only in unplanted soil. After procyanidin amendment, 1409 OTUs were shared by unamended and unamended lettuce, whereas only 49 and 90 OTUs were specific for unamended and amended lettuce, respectively. The two planted soils shared 1171 OTUs and presented 319 and 287 unique OTUs for Manziat and Serail soils, respectively. The two amended planted soils shared 1201 OTUs and presented 279 and 298 unique OTUs for lettuce grown in Manziat and Serail soil, respectively.

For fungi (Figure 5B), in Manziat soil, 216 OTUs were shared by the unplanted and planted soil, 44 OTUs were present only in lettuce plants and 28 OTUs were present only in unplanted soil. After procyanidin amendment, 223 OTUs were shared by unamended and unamended lettuce, whereas only 37 and 11 OTUs were specific to unamended and amended lettuce, respectively. For Serail soil, 279 OTUs were shared by unplanted and planted soil, 29 OTUs were present only in lettuce plants, and 25 OTUs were present only in unplanted soil. After procyanidin amendment, 283 OTUs were shared by unamended and unamended lettuce, whereas only 25 and 26 OTUs were specific for unamended and amended lettuce, respectively. The two planted soils shared 166 OTUs and presented 94 and 142 unique OTUs for Manziat and Serail soils, respectively. The two amended planted soils shared 145 OTUs and presented 89 and 164 unique OTUs for lettuce grown in Manziat and Serail soil, respectively.

### 3.4. Relationship between Microbiome Diversity and Edaphic Variables

**Figure 6:**
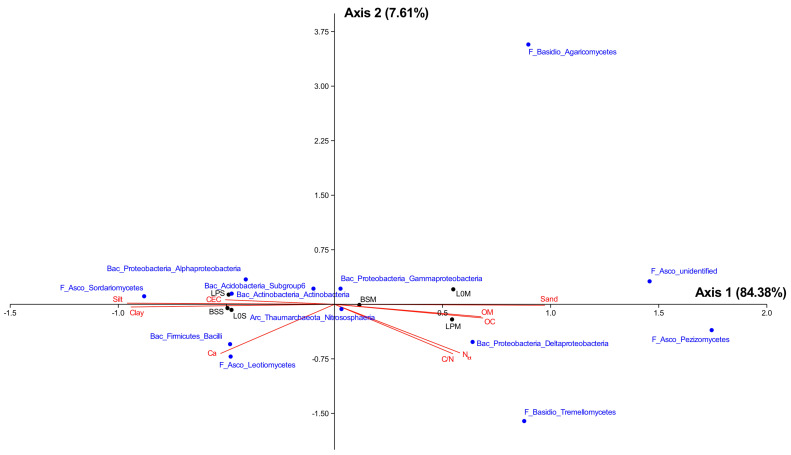
Canonical correspondence analysis (CCA) between nine physicochemical characteristics measured during the experiment (sand, silt, clay, organic carbon (OC), organic matter (OM), total nitrogen (N_tot_), C/N ratio (C/N), calcium (Ca), and cation exchange capacity (CEC)) and the most abundant prokaryotic and fungal phylum/classes (>5% of sequences in their respective dataset). Bac: bacteria; Arc: archaea; F: fungi.

The canonical correspondence analysis explained 84.38 and 7.61% of the variance on the first and the second axes, respectively (Figure 6). This analysis, based on group abundancy, clearly showed a segregation of the samples from Serail soil versus those collected from Manziat soil. In Serail soil, the community composition was mainly related to CEC and silt for the amended lettuce (LPS) and to Ca^2+^ for the unamended lettuce (L0S). In Manziat, it was mostly related to C/N ratio, N_tot_, OM, and OC for the amended lettuce (LPM) and to sand for the unamended lettuce (L0M). Among prokaryotic and fungal communities, Sordariomycetes and Firmicutes were mainly related to CEC and/or Ca^2+^, while Dothideomycetes were related to silt as were Actinobacteria, Planctomycetes, and Leotiomycetes (Figure 6). On the other hand, the C/N ratio and/or N_tot_ were related to the distribution of Agaricomycetes and Saccharomycetes, while Pezizomycetes were related to sand. Other groups such as Thaumarchaeota or Chloroflexi were related to OM and/or OC content.

### 3.5. Prediction of Fungal and Prokaryotic Ecological Functions

Unsurprisingly, among the 3551 KEGG categories inferred from the prokaryotic dataset, numerous categories were different between soils (Manziat versus Serail). Specifically, 56 categories were statistically enriched in Manziat soil, while 55 categories were enriched in Serail soil based on an LDA score >2 (Table S3). Among the 56 categories related to Manziat, five were related to nitrogen metabolism, including *nifHDK* (nitrogen fixation) and *nrfA* (nitrite reductase), while two in Serail were related to the N cycle, namely *narG/narZ* (nitrate reductase) and *nxrA* (nitrate oxidoreductase). In Serail, it should be noted that three categories were related to the biosynthesis of siderophores and nonribosomal peptides and two categories were related to multidrug efflux pump, which were absent in Manziat soil. In contrast, interestingly, procyanidin treatment did not influence the statistically inferred functions.

Among the 427 fungal OTUs, only 280 were functionally inferred (corresponding to 34.43% of fungal richness) and 227 were retained because they fitted both criteria for an acceptable confidence rank and a blast identity >95%. According to trophic modes, saprotrophs were dominant irrespective of the samples (Figure S2). Other trophic modes were also recorded as pathotrophs–saprotrophs–symbiotrophs, pathotrophs, or saprotrophs–symbiotrophs.

### 3.6. Microbial Network Description

**Figure 7:**
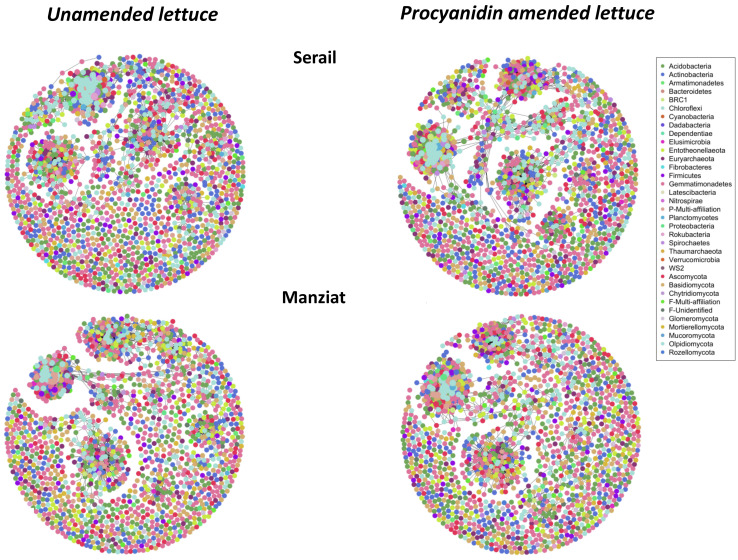
Co-occurrence network of bacterial and fungal microbial community associated with the root-adhering soil retrieved from lettuce plants amended and unamended with procyanidins using Serail or Manziat soils as reservoir.

We used a network inference based on strong and significant correlations to explore the co-occurrences among bacterial and fungal phyla colonizing the RAS of lettuce plants, amended and unamended with procyanidins, grown in Serail and Manziat soils (Figure 7, Table S4). In the RAS of lettuce grown in Manziat soil, there were 1750 and 1714 nodes for unamended and amended soil with procyanidins, respectively. In the RAS of lettuce grown in Serail soil, there were 1766 and 1808 nodes for unamended and amended soil with procyanidins, respectively. The microbial network from unamended lettuce grown in Manziat soil presented 3950 links, whereas that of Serail soil presented 2743 links. After procyanidin amendment, the microbial network of unamended lettuce grown in Manziat soil presented 2637 links, whereas that of Serail soil presented 4484 links (Table S4).

Network complexity, indicated by the number and density of edges, was different between the two soils planted with lettuce (Table S4). The network from the RAS of lettuce grown in Manziat soil was more complex than that of the RAS of lettuce grown in Serail soil without amendment. In contrast, after amendment, the network was less complex in Manziat soil, whereas it was more complex in Serail soil (Table S4 and Figure 7). The average path length (APL) of the amended Manziat soil was significantly higher than the corresponding unamended network (Table S4). For the amended Serail network, the APL was significantly lower than the corresponding unamended network and the amended Manziat soil. This result suggested that the Serail network had “small-world” properties (Table S4).

The microbial network also differed for the two amended and unamended soils with regard to composition. The RAS network of the unamended Serail soil was more enriched in Euryarchaeota, Spirochaetes, Basidiomycetes, and Glomeromycota, while the network of the amended soil was enriched in Entotheonellaeota and Mucoromycota. The RAS network of the unamended Manziat soil had lower proportions of Basidiomycetes and Latescibacteria, while it was enriched in Dependentiae, Aramatomonadetes, and Rozellomycetes compared to the amended Manziat soil. The RAS network of the amended Manziat soil was also enriched with Mucoromycota as observed for the amended Serail soil. Notably, Serail soil networks (unamended and unamended) were more diversified in gungi than Manziat soil.

## 4. Discussion

### 4.1. Soil Type and Edaphic Variables as Driver of Bacterial and Fungal Diversity

The soil environment maintains a high microbial diversity harboring different functions and providing ecosystem services, such as plant growth stimulation and health protection. Manziat and Serail soils harbor a rich microbial community that is clearly distinguishable from each other in its diversity and functions.

The presence of certain phyla has been correlated with certain edaphic variables as demonstrated by Zheng et al. (2019), suggesting that edaphic factors participate in shaping microbial community composition and diversity [60]. Indeed, previous studies have reported a negative response of Acidobacteria to increased available organic C, suggesting that members of this phylum specific for lettuce plants in Serail soil are probably oligotrophic bacteria [61]. As an essential source of energy and nutrients for microorganisms, soil organic matter content (represented by OM and OC or total soil N) plays an important role in shaping microbial communities [62,63]. For instance, organic C and N amendment experiments have revealed significant changes in microbial PLFA composition and fungal/bacterial ratios [63,64,65]. It has been reported that the relative abundance of Actinobacteria increases with the amount of soil C and N pool [66]. This contradicts the typically observed positive relationship, which may be due to differences in SOM quality and accessibility. Deltaproteobacteria were also strongly correlated with total N, corroborating previous research [67,68]. Interestingly, Leotiomycetes and Firmicutes were correlated with Ca^2+^. The effect of Ca^2+^ on microbial communities has been documented in a few studies. Ca^2+^ in soil influences many soil factors, including pH [69]. Indeed, Sridevi et al. (2012) reported that an increase in Ca^2+^ could accompany a change in other soil parameters, thereby resulting in variations in microbial diversity between calcium-amended and reference watershed soils [70].

### 4.2. Procyanidin Amendment Does Not Affect the Diversity and Potential Activity of the Plant Microbiome

In this study, we used procyanidins as denitrification inhibitors. Extensive experimental evidence has demonstrated the effectiveness of these specific compounds in (i) reducing N losses by denitrification and hence increasing the nitrate pool in the soil, which can then be used for plant growth, and (ii) decreasing N_2_O emissions from agricultural soils [32,34,35,36,71]. Our previous in situ studies demonstrated that procyanidins specifically acted on the denitrification process as only denitrification enzyme activity (DEA) was affected after procyanidin amendment [33]. No effect was observed on substrate-induced respiration (SIR) activity nor nitrification activity (NEA). In addition, using the same field experiment, we demonstrated that procyanidin amendment at 210 kg ha^−1^ inhibited denitrification activity and acted on the abundance of denitrifying bacterial communities in both soils. Indeed, whether in loamy sand or sandy clay loam soil, denitrifying communities were less abundant in procyanidin-amended soil than in unamended soil [33]. However, no information is currently available on the impact of procyanidin amendment on nontarget soil microbial diversity and its potential functions. In order to use procyanidins as a sustainable solution to reduce N input and N_2_O emissions in agricultural soils, this molecule should not have any impact on soil fertility and consequently on the diversity and functioning of nontarget soil microbial communities. For example, Kong et al. (2016) demonstrated that soil amended with DMPP at doses consistent with, and 10-fold higher than, those recommended did not exert significant negative impacts on nontarget soil functions or microbial community size or composition after short-term exposure [72]. In this study, we found that procyanidin amendment did not affect bacterial and fungal diversity in both soil types. In addition, procyanidin amendment did not influence statistically inferred prokaryotic and eukaryotic functions. Procyanidin amendment at 210 kg ha^−1^ corresponds to a dose with a significant and specific impact on denitrification activity compared to the lower doses (8, 42, and 83 kg ha^−1^) tested in Galland et al. [35]. Our hypothesis was that procyanidins at 210 kg ha^−1^ would be more bioavailable in the soil than less concentrated procyanidins and hence in better contact with microorganisms. The fact that procyanidin amendment at 210 kg ha^−1^ did not affect microbial diversity ensures the environmental safety of procyanidin intervention in soils under field conditions.

### 4.3. Procyanidin Amendment Modifies Microbial Network

Under natural conditions, Manziat soil exhibited a more complex and stable network comprising more interacting species than Serail soil. Procyanidin amendment decreased the order organization of Manziat soil, while it enhanced that of Serail soil. Interestingly, procyanidin amendment improved lettuce growth in both soils [34], suggesting that the robustness of the microbial network may not be systematically related to plant health. The network complexity and stability do not necessarily reflect the beneficial properties of the microbiome as certain deleterious microbial species may be highly connected to other microorganisms.

## 5. Conclusions

In order to use procyanidins as a sustainable solution to reduce N input and N_2_O emissions in agricultural soils, this molecule should not have any impact on soil fertility and consequently the diversity and functioning of nontarget soil microbial communities. In this study, we demonstrated that procyanidin amendment to lettuce plants had no impact on soil microbial diversity. Indeed, bacterial and fungal diversity did not change with procyanidin amendment and only changed according to soil type and edaphic variables. Therefore, procyanidins may represent an alternative to the use of N fertilizers, thus reducing N input and N_2_O emissions.

## Figures and Tables

**Table 1:** Number of prokaryotic (**A**) and fungal (**B**) operational taxonomic units (OTUs), species richness (Chao1 index) and diversity (Simpson, Shannon, and evenness) indices obtained bulk soil in Manziat and Serail (BSM and BSS, respectively) for lettuce planted with and without procyanidin amendment in Manziat soil (L0M and LPM) and in Serail soil (L0S and LPS).

(**A**)												
	**BSM**		**L0M**		**LPM**		**BSS**		**L0S**		**LPS**	
	**Mean**	**SD**	**Mean**	**SD**	**Mean**	**SD**	**Mean**	**SD**	**Mean**	**SD**	**Mean**	**SD**
**Taxa** **Number**	1138.7	89.0	1162.3	38.0	1162.7	34.2	1066.3	80.7	1172.0	36.5	1183.7	24.1
**Simpson**	1.0	0.0	1.0	0.0	1.0	0.0	1.0	0.0	1.0	0.0	1.0	0.0
**Shannon**	6.2	0.2	6.3	0.0	6.2	0.0	5.5	0.8	6.2	0.0	6.2	0.1
**Evenness**	0.4	0.1	0.5	0.0	0.4	0.0	0.3	0.2	0.4	0.0	0.4	0.1
**Chao-1**	1350.0	74.1	1374.7	28.7	1380.0	46.5	1357.7	25.8	1353.3	33.2	1371.3	29.6
(**B**)												
	**BSM**		**L0M**		**LPM**		**BSS**		**L0S**		**LPS**	
	**Mean**	**SD**	**Mean**	**SD**	**Mean**	**SD**	**Mean**	**SD**	**Mean**	**SD**	**Mean**	**SD**
**Taxa** **Number**	193.7	5.1	202.3	3.5	186.3	16.8	245.7	11.9	249.7	4.9	242.0	24.9
**Simpson**	0.9	0.0	0.9	0.0	0.9	0.0	0.9	0.0	0.9	0.0	0.9	0.0
**Shannon**	3.4	0.3	3.4	0.1	3.2	0.1	3.6	0.2	3.6	0.1	3.6	0.4
**Evenness**	0.2	0.0	0.1	0.0	0.1	0.0	0.2	0.0	0.2	0.0	0.2	0.1
**Chao-1**	205.2	7.7	216.9	6.6	195.7	18.0	261.9	13.8	283.4	14.4	260.7	10.7

**Table 2:** Effect of lettuce, procyanidin amendment, and soil on OTU abundance (**A**) and richness (**B**) of 16S rRNA gene and fungal ITS. The analysis was performed using a permutational multivariate analysis of variance (PERMANOVA).

**(A) Abundance of OTUs**								
	**Prokaryotic 16S rRNA Gene**				**Fungal ITS**			
	Df	F	*p* value	R^2^	Df	F	*p* value	R^2^
**Effect**								
Plant	1	1.40	0.184	0.048	1	1.41	0.138	0.039
Treatment	1	0.52	0.875	0.018	1	0.96	0.357	0.027
Soil	1	13.31	**0.001**	**0.457**	1	19.28	**0.001**	**0.541**
**Effect Interactions**								
Plant*Soil	1	1.32	0.231	0.045	1	1.15	0.294	0.032
Treatment*Soil	1	0.54	0.853	0.019	1	0.84	0.460	0.235
**Residuals**	12			0.412	12			0.337
**(B) Richness of OTUs**								
	**Prokaryotic 16S rRNA Gene**				**Fungal ITS**			
	Df	F	*p* value	R^2^	Df	F	*p* value	R^2^
**Effect**								
Plant	1	1.33	0.156	0.033	1	1.88	0.154	0.024
Treatment	1	0.72	0.447	0.018	1	1.24	0.280	0.016
Soil	1	24.56	**0.001**	**0.609**	1	62.60	**0.001**	**0.790**
**Effect Interactions**								
Plant*Treatment*Soil	1	0.87	0.445	0.043	1	0.78	0.506	0.020
**Residuals**	12			0.198	12			0.151

## Data Availability

The sequence data generated in this study is deposited at EMBL-ENA public database (PRJEB41569 for the prokaryotic 16S rRNA gene dataset and PRJEB41574 for the fungal ITS gene dataset).

## Supplementary Materials

The following are available online at https://www.mdpi.com/article/10.3390/microorganisms9071406/s1, Table S1: Effect of lettuce and procyanidins amendment on 16S rRNA gene abundance in Manziat (A) and Serail (B). The analysis was performed using a Permutational Multivariate Analysis of Variance (PERMANOVA), Table S2: Relative abundance of Archaea and Bacteria in bulk soil in Manziat and Serail (BSM and BSS, respectively), for Lettuce-planted conditions without and with procyanidins amendment in Manziat soil (L0M and LPM) and in Serail soil (L0S and LPS), Table S3: Prokaryotic functional inference and KEGG assignment. Statically enriched functions in Manziat *versus* Serail were determined so as LDA score > 2, Table S4: Global network statistics for microbial networks from the root-adhering soil of lettuce plants unamended and amended with procyanidins, Figure S1: Rarefaction curves for each sample were calculated on prokaryotic 16S rRNA gene (A) and fungal ITS datasets (B), Figure S2: Trophic mode of fungal community retrieved form amended and unamended Manziat and Serail soils with procyanidins.

## References

[1] Boserup E. (2017). The Conditions of Agricultural Growth: The Economics of Agrarian Change under Population Pressure.

[2] Tilman D., Cassman K.G., Matson P.A., Naylor R., Polasky S. (2002). Agricultural sustainability and intensive production practices. Nature.

[3] Bloom A.J. (2015). The increasing importance of distinguishing among plant nitrogen sources. Curr. Opin. Plant Biol..

[4] Ronen E. (2016). Micro-elements in agriculture. Pract. Hydroponics Greenh..

[5] Kirchmann H., Johnston A.J., Bergström L.F. (2002). Possibilities for reducing nitrate leaching from agricultural land. AMBIO J. Hum. Environ..

[6] Beaudoin N., Saad J.K., Van Laethem C., Machet J.M., Maucorps J., Mary B. (2005). Nitrate leaching in intensive agriculture in Northern France: Effect of farming practices, soils and crop rotations. Agric. Ecosyst. Environ..

[7] Cassman K.G., Dobermann A., Walters D.T. (2002). Agroecosystems, nitrogen-use efficiency, and nitrogen management. AMBIO J. Hum. Environ..

[8] Di H.J., Cameron K.C. (2002). Nitrate leaching in temperate agroecosystems: Sources, factors and mitigating strategies. Nutr. Cycl. Agroecosyst..

[9] Bouwman A.F., Boumans L.J.M., Batjes N.H. (2002). Estimation of global NH3 volatilization loss from synthetic fertilizers and animal manure applied to arable lands and grasslands. Glob. Biogeochem. Cycles.

[10] Strebel O., Duynisveld W.H.M., Böttcher J. (1989). Nitrate pollution of groundwater in Western Europe. Agric. Ecosyst. Environ..

[11] Lewis W.M., Wurtsbaugh W.A., Paerl H.W. (2011). Rationale for control of anthropogenic nitrogen and phosphorus to reduce eutrophication of inland waters. Environ. Sci. Technol..

[12] Kononen K. (2001). Eutrophication, harmful algal blooms and species diversity in phytoplankton communities: Examples from the Baltic Sea. AMBIO J. Hum. Environ..

[13] Yang X., Wu X., Hao H., He Z. (2008). Mechanisms and assessment of water eutrophication. J. Zhejiang Univ. Sci. B.

[14] Zumft W.G. (1997). Cell biology and molecular basis of denitrification. Microbiol. Mol. Biol. Rev..

[15] Oenema O., Witzke H.P., Klimont Z., Lesschen J.P., Velthof G.L. (2009). Integrated assessment of promising measures to decrease nitrogen losses from agriculture in EU-27. Agric. Ecosyst. Environ..

[16] Galloway J.N., Townsend A.R., Erisman J.W., Bekunda M., Cai Z., Freney J.R., Martinelli L.A., Seitzinger S.P., Sutton M.A. (2008). Transformation of the nitrogen cycle: Recent trends, questions, and potential solutions. Science.

[17] Ravishankara A.R., Daniel J.S., Portmann R.W. (2009). Nitrous oxide (N_2_O): The dominant ozone-depleting substance emitted in the 21st century. Science.

[18] Akiyama H., Yan X., Yagi K. (2010). Evaluation of effectiveness of enhanced-efficiency fertilizers as mitigation options for N2O and NO emissions from agricultural soils: Meta-analysis. Glob. Change Biol..

[19] Santamaria P., Elia A., Serio F., Gonnella M., Parente A. (1999). Comparison between nitrate and ammonium nutrition in fennel, celery, and Swiss chard. J. Plant Nutr..

[20] Santamaria P. (2006). Nitrate in vegetables: Toxicity, content, intake and EC regulation. J. Sci. Food Agric..

[21] Green L.C., De Luzuriaga K.R., Wagner D.A., Rand W., Istfan N., Young V.R., Tannenbaum S.R. (1981). Nitrate biosynthesis in man. Proc. Natl. Acad. Sci. USA.

[22] Van Velzen A.G., Sips A.J., Schothorst R.C., Lambers A.C., Meulenbelt J. (2008). The oral bioavailability of nitrate from nitrate-rich vegetables in humans. Toxicol. Lett..

[23] Du Jardin P. (2015). Plant biostimulants: Definition, concept, main categories and regulation. Sci. Hortic..

[24] Gilsanz C., Báez D., Misselbrook T.H., Dhanoa M.S., Cárdenas L.M. (2016). Development of emission factors and efficiency of two nitrification inhibitors, DCD and DMPP. Agric. Ecosyst. Environ..

[25] Liu C., Wang K., Zheng X. (2013). Effects of nitrification inhibitors (DCD and DMPP) on nitrous oxide emission, crop yield and nitrogen uptake in a wheat-maize cropping system. Biogeosciences.

[26] Nardi P., Laanbroek H.J., Nicol G.W., Renella G., Cardinale M., Pietramellara G., Weckwerth W., Trinchera A., Ghatak A., Nannipieri P. (2020). Biological nitrification inhibition in the rhizosphere: Determining interactions and impact on microbially mediated processes and potential applications. FEMS Microbiol. Rev..

[27] Abalos D., Jeffery S., Sanz-Cobena A., Guardia G., Vallejo A. (2014). Meta-analysis of the effect of urease and nitrification inhibitors on crop productivity and nitrogen use efficiency. Agric. Ecosyst. Environ..

[28] Pathak H., Bhatia A., Prasad S., Singh S., Kumar S., Jain M.C., Kumar U. (2002). Emission of nitrous oxide from rice-wheat systems of Indo-Gangetic plains of India. Environ. Monit. Assess..

[29] Meijide A., Díez J.A., Sánchez-Martín L., López-Fernández S., Vallejo A. (2007). Nitrogen oxide emissions from an irrigated maize crop amended with treated pig slurries and composts in a Mediterranean climate. Agric. Ecosyst. Environ..

[30] Di H.J., Cameron K.C. (2012). How does the application of different nitrification inhibitors affect nitrous oxide emissions and nitrate leaching from cow urine in grazed pastures?. Soil Use Manag..

[31] Tedeschi A., De Marco A., Polimeno F., Di Tommasi P., Maglione G., Ottaiano L., Arena C., Magliulo V., Vitale L. (2021). Effects of the fertilizer added with DMPP on soil nitrous oxide emissions and microbial functional diversity. Agriculture.

[32] Bardon C., Poly F., Piola F., Pancton M., Comte G., Meiffren G., el Zahar Haichar F. (2016). Mechanism of biological denitrification inhibition: Procyanidins induce an allosteric transition of the membrane-bound nitrate reductase through membrane alteration. FEMS Microbiol. Ecol..

[33] Bardon C., Poly F., el Zahar Haichar F., Le Roux X., Simon L., Meiffren G., Comte G., Rouifed S., Piola F. (2017). Biological denitrification inhibition (BDI) with procyanidins induces modification of root traits, growth and N status in *Fallopia* x *bohemica*. Soil Biol. Biochem..

[34] Galland W., Piola F., Mathieu C., Bouladra L., Simon L., el Zahar Haichar F. (2020). Does biological denitrification inhibition (BDI) in the field induce an increase in plant growth and nutrition in *Apium graveolens* L. grown for a long period?. Microorganisms.

[35] Galland W., Piola F., Burlet A., Mathieu C., Nardy M., Poussineau S., Blazère L., Gervaix J., Puijalon S., Simon L. (2019). Biological denitrification inhibition (BDI) in the field: A strategy to improve plant nutrition and growth. Soil Biol. Biochem..

[36] Galland W., el Zahar Haichar F., Czarnes S., Mathieu C., Demorge J.-L., Simon L., Puijalon S., Piola F. (2021). Biological inhibition of denitrification (BDI) in the field: Effect on plant growth in two different soils. Appl. Soil Ecol..

[37] Preece D.A. (1990). RA fisher and experimental design: A review. Biometrics.

[38] Hénin S. (1976). Cours de Physique du Sol: Texture-Structure-Aération.

[39] Despujols J., Station d’Experimentation et d’Information R.A.L. (1997). Control of nitrate level in autumn greenhouse lettuce. Infos CTIFL Fr..

[40] Caporaso J.G., Lauber C.L., Walters W.A., Berg-Lyons D., Lozupone C.A., Turnbaugh P.J., Fierer N., Knight R. (2011). Global patterns of 16S RRNA diversity at a depth of millions of sequences per sample. Proc. Natl. Acad. Sci. USA.

[41] Wang Y., Qian P.-Y. (2009). Conservative fragments in bacterial 16S RRNA genes and primer design for 16S ribosomal DNA amplicons in metagenomic studies. PLoS ONE.

[42] Ihrmark K., Bödeker I.T.M., Cruz-Martinez K., Friberg H., Kubartova A., Schenck J., Strid Y., Stenlid J., Brandström-Durling M., Clemmensen K.E. (2012). New primers to amplify the fungal ITS2 region-evaluation by 454-sequencing of artificial and natural communities. FEMS Microbiol. Ecol..

[43] White T.J., Burns T.D., Lee S.B., Taylor J.W. (1990). Amplification and direct sequencing of fungal ribosomal RNA genes for phylogenetics. PCR Protoc. Appl. Lab. Man..

[44] Schoch C.L., Seifert K.A., Huhndorf S., Robert V., Spouge J.L., Levesque C.A., Chen W., Consortium F.B. (2012). Nuclear ribosomal internal transcribed spacer (ITS) region as a universal DNA barcode marker for fungi. Proc. Natl. Acad. Sci. USA.

[45] Rognes T., Flouri T., Nichols B., Quince C., Mahé F. (2016). VSEARCH: A versatile open source tool for metagenomics. PeerJ.

[46] Mahé F., Rognes T., Quince C., de Vargas C., Dunthorn M. (2014). Swarm: Robust and fast clustering method for amplicon-based studies. PeerJ.

[47] Bokulich N.A., Subramanian S., Faith J.J., Gevers D., Gordon J.I., Knight R., Mills D.A., Caporaso J.G. (2013). Quality-filtering vastly improves diversity estimates from illumina amplicon sequencing. Nat. Methods.

[48] Wang Q., Garrity G.M., Tiedje J.M., Cole J.R. (2007). Naive Bayesian classifier for rapid assignment of RRNA sequences into the new bacterial taxonomy. Appl. Environ. Microbiol..

[49] Camacho C., Coulouris G., Avagyan V., Ma N., Papadopoulos J., Bealer K., Madden T.L. (2009). BLAST+: Architecture and applications. BMC Bioinform..

[50] Pruesse E., Quast C., Knittel K., Fuchs B.M., Ludwig W., Peplies J., Glöckner F.O. (2007). SILVA: A comprehensive online resource for quality checked and aligned ribosomal RNA sequence data compatible with ARB. Nucleic Acids Res..

[51] Kõljalg U., Nilsson R.H., Abarenkov K., Tedersoo L., Taylor A.F.S., Bahram M., Bates S.T., Bruns T.D., Bengtsson-Palme J., Callaghan T.M. (2013). Towards a unified paradigm for sequence-based identification of fungi. Mol. Ecol..

[52] Escudié F., Auer L., Bernard M., Mariadassou M., Cauquil L., Vidal K., Maman S., Hernandez-Raquet G., Combes S., Pascal G. (2017). FROGS: Find, rapidly, OTUs with galaxy solution. Bioinformatics.

[53] Anderson M.J. (2001). A new method for non-parametric multivariate analysis of variance. Austral. Ecol..

[54] Ramette A. (2007). Multivariate analyses in microbial ecology. FEMS Microbiol. Ecol..

[55] Aßhauer K.P., Meinicke P. On the estimation of metabolic profiles in metagenomics. Proceedings of the German Conference on Bioinformatics 2013.

[56] Segata N., Izard J., Waldron L., Gevers D., Miropolsky L., Garrett W.S., Huttenhower C. (2011). Metagenomic biomarker discovery and explanation. Genome Biol..

[57] Nguyen N.H., Song Z., Bates S.T., Branco S., Tedersoo L., Menke J., Schilling J.S., Kennedy P.G. (2016). FUNGuild: An open annotation tool for parsing fungal community datasets by ecological guild. Fungal Ecol..

[58] Tedersoo L., Bahram M., Põlme S., Kõljalg U., Yorou N.S., Wijesundera R., Ruiz L.V., Vasco-Palacios A.M., Thu P.Q., Suija A. (2014). Global diversity and geography of soil fungi. Science.

[59] Layeghifard M., Hwang D.M., Guttman D.S. (2018). Constructing and analyzing microbiome networks in R. Microbiome Analysis.

[60] Zheng Q., Hu Y., Zhang S., Noll L., Böckle T., Dietrich M., Herbold C.W., Eichorst S.A., Woebken D., Richter A. (2019). Soil multifunctionality is affected by the soil environment and by microbial community composition and diversity. Soil Biol. Biochem..

[61] Fierer N., Bradford M.A., Jackson R.B. (2007). Toward an ecological classification of soil bacteria. Ecology.

[62] Burns K.N., Bokulich N.A., Cantu D., Greenhut R.F., Kluepfel D.A., O’Geen A.T., Strauss S.L., Steenwerth K.L. (2016). Vineyard soil bacterial diversity and composition revealed by 16S RRNA genes: Differentiation by vineyard management. Soil Biol. Biochem..

[63] Drenovsky R.E., Vo D., Graham K.J., Scow K.M. (2004). Soil water content and organic carbon availability are major determinants of soil microbial community composition. Microb. Ecol..

[64] Ng E.-L., Patti A.F., Rose M.T., Schefe C.R., Wilkinson K., Smernik R.J., Cavagnaro T.R. (2014). Does the chemical nature of soil carbon drive the structure and functioning of soil microbial communities?. Soil Biol. Biochem..

[65] Zhou Z., Wang C., Zheng M., Jiang L., Luo Y. (2017). Patterns and mechanisms of responses by soil microbial communities to nitrogen addition. Soil Biol. Biochem..

[66] Li Y., Chapman S.J., Nicol G.W., Yao H. (2018). Nitrification and nitrifiers in acidic soils. Soil Biol. Biochem..

[67] Ling N., Chen D., Guo H., Wei J., Bai Y., Shen Q., Hu S. (2017). Differential responses of soil bacterial communities to long-term N and P inputs in a semi-arid steppe. Geoderma.

[68] Zhang X., Chen Q., Han X. (2013). Soil bacterial communities respond to mowing and nutrient addition in a steppe ecosystem. PLoS ONE.

[69] Kim J.M., Roh A.-S., Choi S.-C., Kim E.-J., Choi M.-T., Ahn B.-K., Kim S.-K., Lee Y.-H., Joa J.-H., Kang S.-S. (2016). Soil PH and electrical conductivity are key edaphic factors shaping bacterial communities of greenhouse soils in Korea. J. Microbiol..

[70] Sridevi G., Minocha R., Turlapati S.A., Goldfarb K.C., Brodie E.L., Tisa L.S., Minocha S.C. (2012). Soil bacterial communities of a calcium-supplemented and a reference watershed at the Hubbard Brook Experimental Forest (HBEF), New Hampshire, USA. FEMS Microbiol. Ecol..

[71] Kong X., Duan Y., Schramm A., Eriksen J., Petersen S.O. (2016). 3,4-dimethylpyrazole phosphate (DMPP) reduces activity of ammonia oxidizers without adverse effects on non-target soil microorganisms and functions. Appl. Soil Ecol..

[72] Bardon C., Piola F., Bellvert F., el Zahar Haichar F., Comte G., Meiffren G., Pommier T., Puijalon S., Tsafack N., Poly F. (2014). Evidence for biological denitrification inhibition (BDI) by plant secondary metabolites. New Phytol..

