# Supplementary material for: Effects of the Denitrification Inhibitor “Procyanidins” on the Diversity, Interactions, and Potential Functions of Rhizosphere-Associated Microbiome
Source: Microorganisms. 2021 Jun 29;9(7):1406. doi: 10.3390/microorganisms9071406 (PMC8306639; doi:10.3390/microorganisms9071406)
Supplement: Supplementary file 1 [file microorganisms-09-01406-s001.zip › microorganisms-1269648-supplementary.zip!/Figure S1.pdf]

A. B.

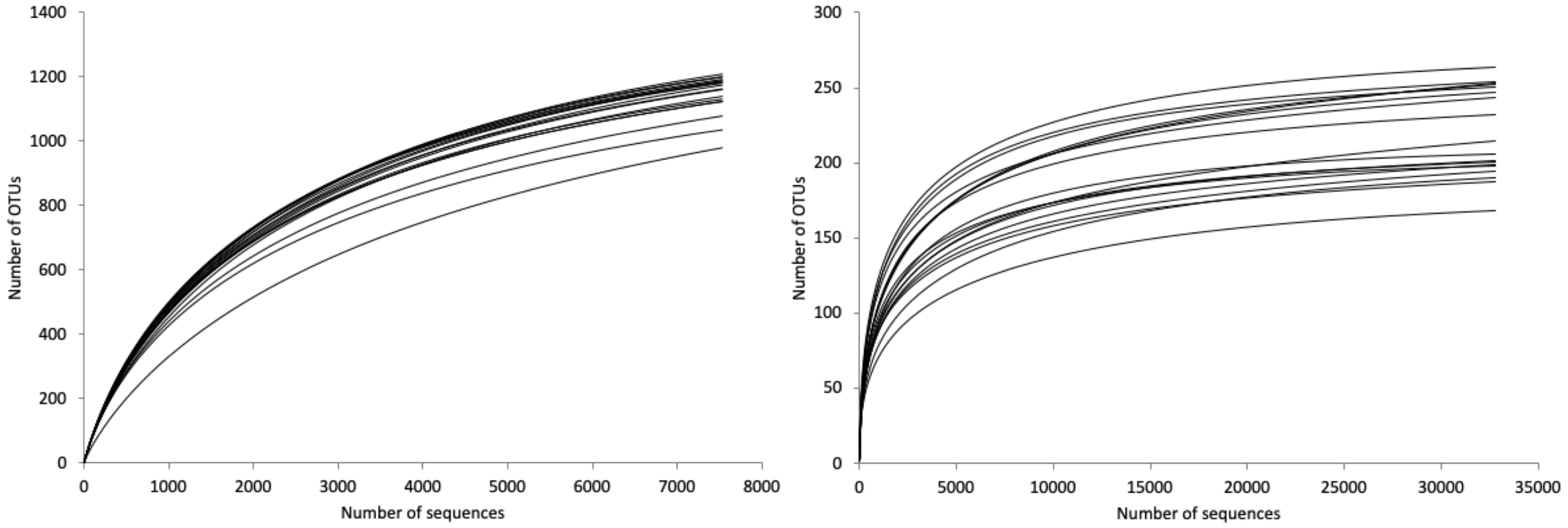

1400
1200
1000
800
600
400
200
0
Number of OTUs
0
1000
2000
3000
4000
5000
6000
7000
8000
Number of sequences
300
250
200
150
100
50
0
Number of OTUs
0
5000
10000
15000
20000
25000
30000
35000
Number of sequences
