# Supplementary material for: Effects of the Denitrification Inhibitor “Procyanidins” on the Diversity, Interactions, and Potential Functions of Rhizosphere-Associated Microbiome
Source: Microorganisms. 2021 Jun 29;9(7):1406. doi: 10.3390/microorganisms9071406 (PMC8306639; doi:10.3390/microorganisms9071406)
Supplement: Supplementary file 1 [file microorganisms-09-01406-s001.zip › microorganisms-1269648-supplementary.zip!/Figure S2.pdf]

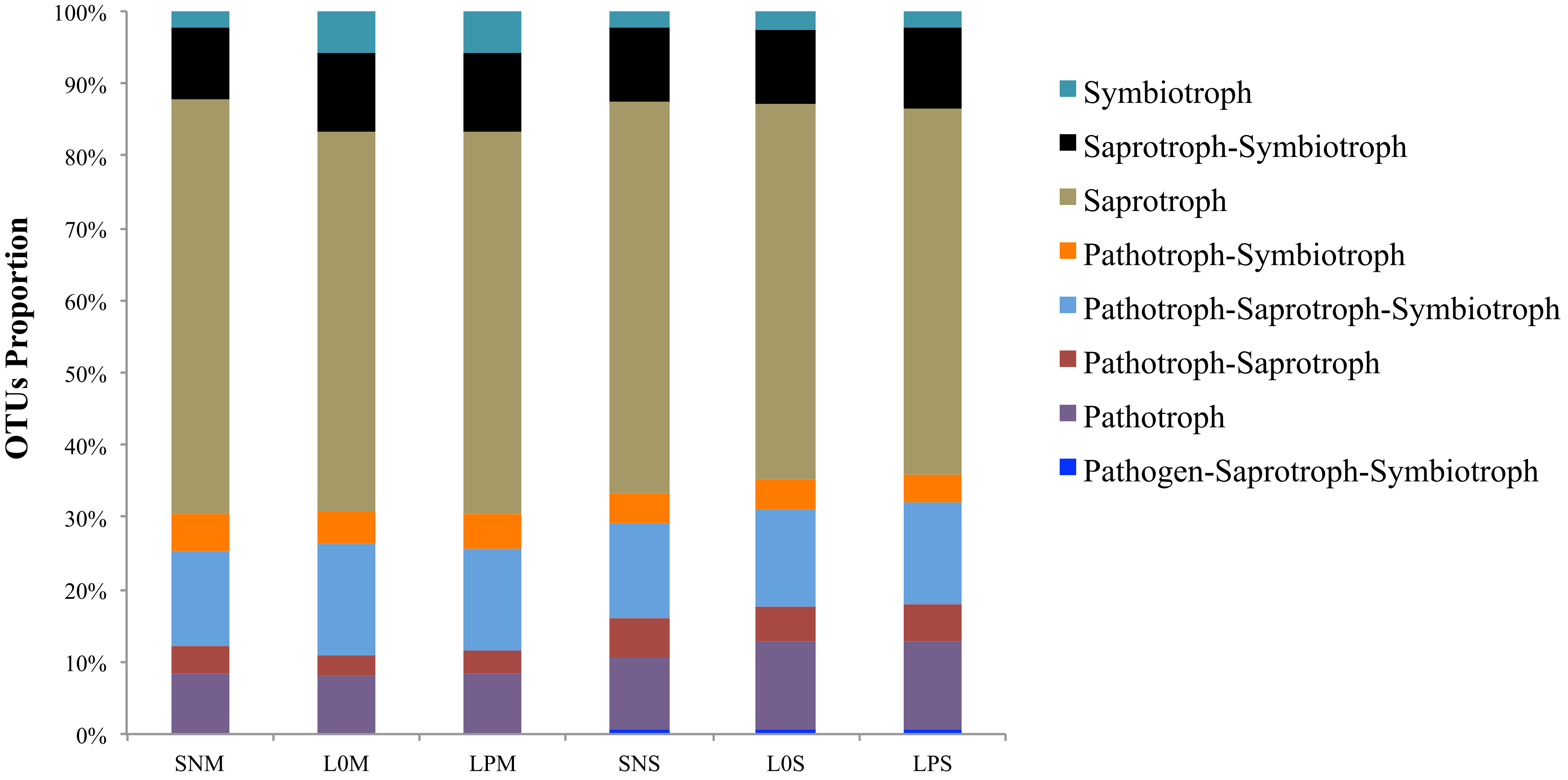

OTUs Proportion
100%
90%
80%
70%
60%
50%
40%
30%
20%
10%
0%
SNM
L0M
LPM
SNS
L0S
LPS
Symbiotroph
Saprotroph-Symbiotroph
Saprotroph
Pathotroph-Symbiotroph
Pathotroph-Saprotroph-Symbiotroph
Pathotroph-Saprotroph
Pathotroph
Pathogen-Saprotroph-Symbiotroph
