# Supplementary material for: Effects of the Denitrification Inhibitor “Procyanidins” on the Diversity, Interactions, and Potential Functions of Rhizosphere-Associated Microbiome
Source: Microorganisms. 2021 Jun 29;9(7):1406. doi: 10.3390/microorganisms9071406 (PMC8306639; doi:10.3390/microorganisms9071406)
Supplement: Supplementary file 1 [file microorganisms-09-01406-s001.zip › microorganisms-1269648-supplementary.zip!/Supplementary data.pdf]

Supplementary data:

Table S1. Effect of lettuce and procyanidins amendment on 16S rRNA gene abundance in Manziat (A) and Serail (B). The analysis was performed using a Permutational Multivariate Analysis of Variance (PERMANOVA).

Table S2. Relative abundance of Archaea and Bacteria in bulk soil in Manziat and Serail (BSM and BSS, respectively), for Lettuce-planted conditions without and with procyanidins amendment in Manziat soil (L0M and LPM) and in Serail soil (L0S and LPS).

Table S3. Prokaryotic functional inference and KEGG assignment. Statically enriched functions in Manziat *versus* Serail were determined so as LDA score > 2.

Table S4. Global network statistics for microbial networks from the root-adhering soil of lettuce plants unamended and amended with procyanidins.

Figure S1. Rarefaction curves for each sample were calculated on prokaryotic 16S rRNA gene (A) and fungal ITS datasets (B).

Figure S2. Trophic modes of fungal community.
