# Supplementary material for: Effects of the Denitrification Inhibitor “Procyanidins” on the Diversity, Interactions, and Potential Functions of Rhizosphere-Associated Microbiome
Source: Microorganisms. 2021 Jun 29;9(7):1406. doi: 10.3390/microorganisms9071406 (PMC8306639; doi:10.3390/microorganisms9071406)
Supplement: Supplementary file 1 [file microorganisms-09-01406-s001.zip › microorganisms-1269648-tex.zip!/Definitions/logo-ccby-eps-converted-to.pdf]

CC BY
