# Supplementary material for: Effects of the Denitrification Inhibitor “Procyanidins” on the Diversity, Interactions, and Potential Functions of Rhizosphere-Associated Microbiome
Source: Microorganisms. 2021 Jun 29;9(7):1406. doi: 10.3390/microorganisms9071406 (PMC8306639; doi:10.3390/microorganisms9071406)
Supplement: Supplementary file 1 [file microorganisms-09-01406-s001.zip › microorganisms-1269648-tex.zip!/Definitions/microorganisms-1269648-g001.pdf]

A)

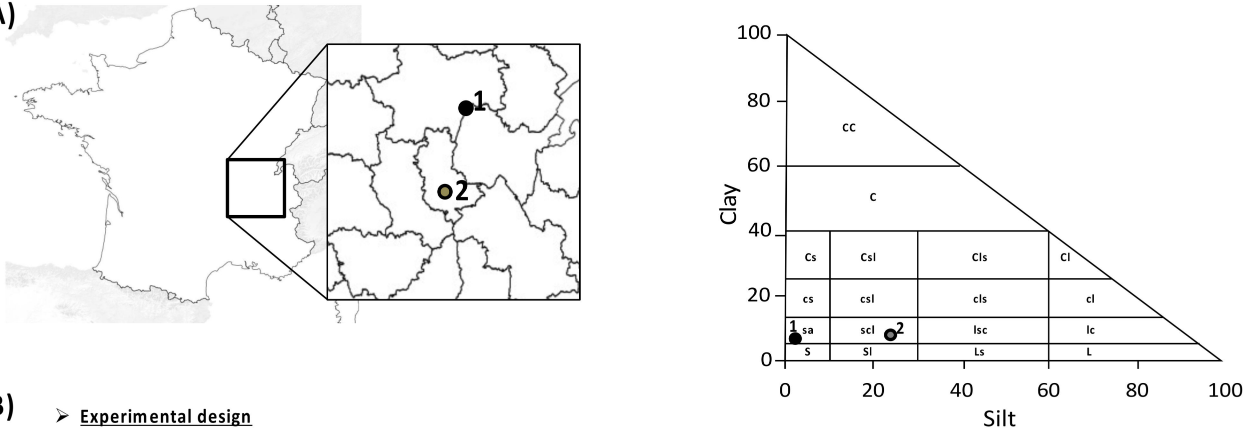

B)

- **Experimental design**

Serail & Manziat soils

Lettuce soil unamended with procyanidins	Lettuce soil amended with procyanidins at 210 kg ha^{-1}	Unplanted soil
80 Lettuces plot 1	80 Lettuces plot 1	Unplanted soil plot 1
80 Lettuces plot 2	80 Lettuces plot 2	Unplanted soil plot 2
80 Lettuces plot 3	80 Lettuces plot 3	Unplanted soil plot 3
80 Lettuces plot 4	80 Lettuces plot 4	Unplanted soil plot 4

- **Harvesting strategy**

**Soil analysis in duplicates**	Pool of rhizospheric soil from 4 lettuces per plots X 2 plots	Samples of bulk soil from 2 plots per treatment
**DNA extraction for microbial diversity analysis in triplicates**	Pool of rhizospheric soil from 4 lettuces per plots X 3 plots	Samples of bulk soil from 3 plots per treatment
