# Supplementary material for: Effects of the Denitrification Inhibitor “Procyanidins” on the Diversity, Interactions, and Potential Functions of Rhizosphere-Associated Microbiome
Source: Microorganisms. 2021 Jun 29;9(7):1406. doi: 10.3390/microorganisms9071406 (PMC8306639; doi:10.3390/microorganisms9071406)
Supplement: Supplementary file 1 [file microorganisms-09-01406-s001.zip › microorganisms-1269648-tex.zip!/Definitions/microorganisms-1269648-g002.pdf]

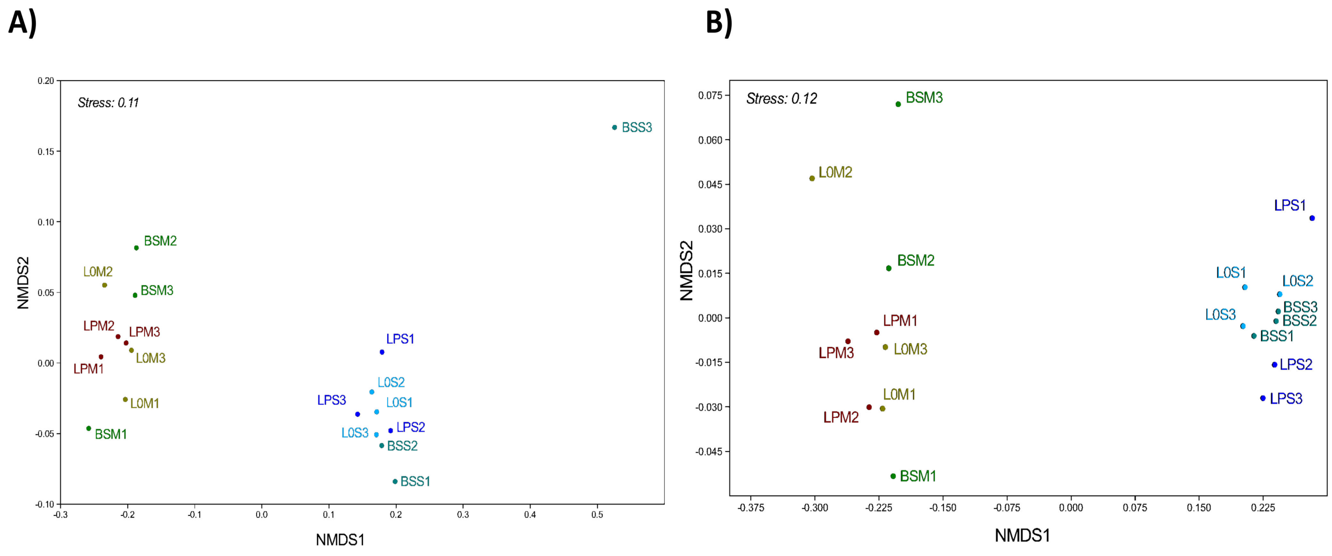
A)
Stress: 0.11
BSS3
BSM2
L0M2
BSM3
LPM2
LPM3
LPM1
L0M3
LPS1
L0S2
LPS3
L0S1
L0M1
L0S3
LPS2
BSM1
BSS2
BSS1
NMDS2
NMDS1
0.20
0.15
0.10
0.05
0.00
-0.05
-0.10
-0.3
-0.2
-0.1
0.0
0.1
0.2
0.3
0.4
0.5
B)
Stress: 0.12
BSM3
L0M2
LPS1
BSM2
L0S1
L0S2
BSS3
L0S3
BSS2
LPM1
BSS1
LPM3
L0M3
LPS2
L0M1
LPS3
LPM2
BSM1
NMDS2
NMDS1
0.075
0.060
0.045
0.030
0.015
0.000
-0.015
-0.030
-0.045
-0.375
-0.300
-0.225
-0.150
-0.075
0.000
0.075
0.150
0.225
