# Supplementary material for: Effects of the Denitrification Inhibitor “Procyanidins” on the Diversity, Interactions, and Potential Functions of Rhizosphere-Associated Microbiome
Source: Microorganisms. 2021 Jun 29;9(7):1406. doi: 10.3390/microorganisms9071406 (PMC8306639; doi:10.3390/microorganisms9071406)
Supplement: Supplementary file 1 [file microorganisms-09-01406-s001.zip › microorganisms-1269648-tex.zip!/Definitions/microorganisms-1269648-g003.pdf]

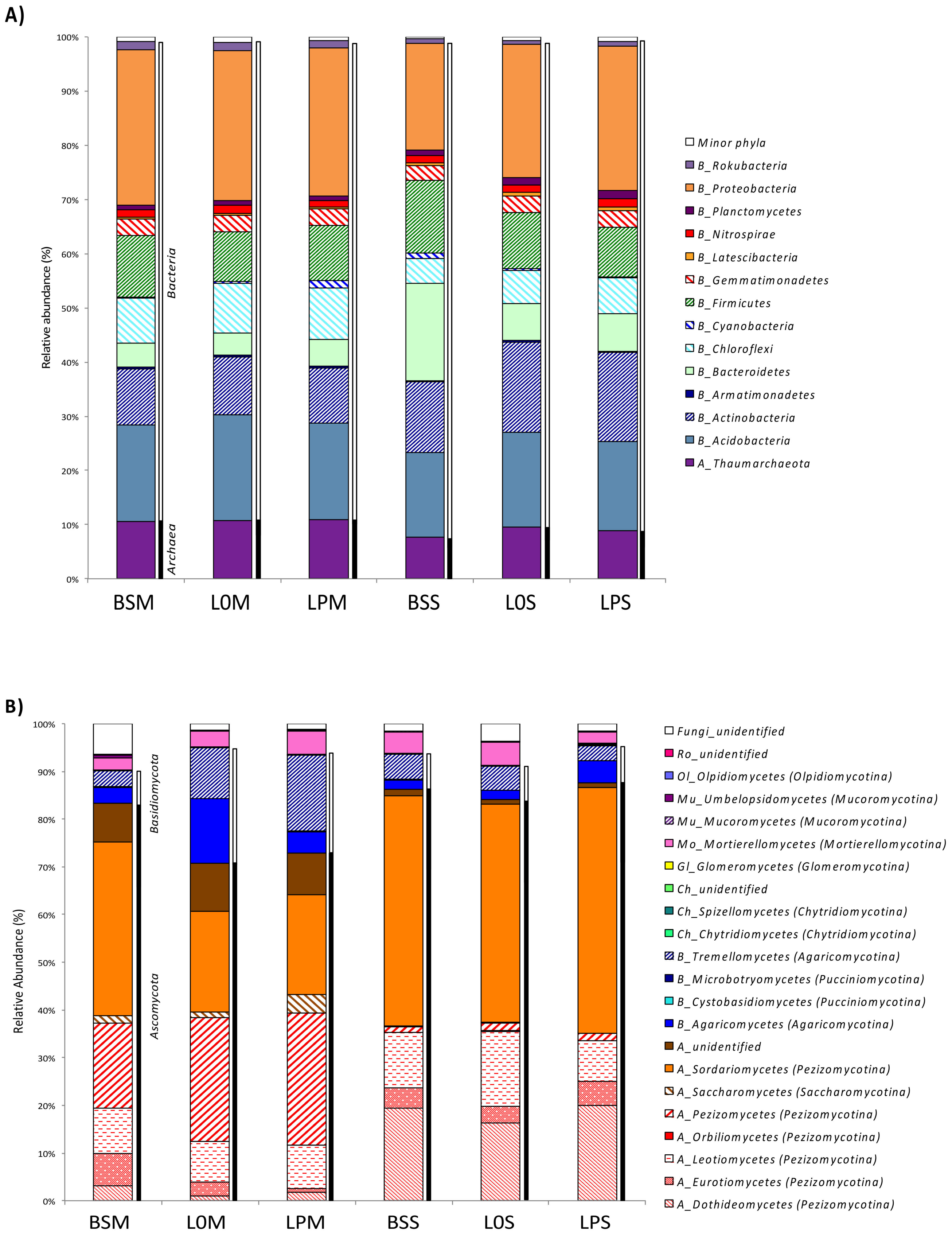
A)
Relative abundance (%)
100%
90%
80%
70%
60%
50%
40%
30%
20%
10%
0%
Bacteria
Archaea
BSM
L0M
LPM
BSS
L0S
LPS
Minor phyla
B_Rokubacteria
B_Proteobacteria
B_Planctomycetes
B_Nitrospirae
B_Latescibacteria
B_Gemmatimonadetes
B_Firmicutes
B_Cyanobacteria
B_Chloroflexi
B_Bacteroidetes
B_Armatimonadetes
B_Actinobacteria
B_Acidobacteria
A_Thaumarchaeota
B)
Relative Abundance (%)
Basidiomycota
Ascomycota
Fungi_unidentified
Ro_unidentified
Ol_Olpidiomycetes (Olpidiomycotina)
Mu_Umbelopsidomycetes (Mucoromycotina)
Mu_Mucoromycetes (Mucoromycotina)
Mo_Mortierellomycetes (Mortierellomycotina)
Gl_Glomeromycetes (Glomeromycotina)
Ch_unidentified
Ch_Spizellomycetes (Chytridiomycotina)
Ch_Chytridiomycetes (Chytridiomycotina)
B_Tremellomycetes (Agaricomycotina)
B_Microbotryomycetes (Pucciniomycotina)
B_Cystobasidiomycetes (Pucciniomycotina)
B_Agaricomycetes (Agaricomycotina)
A_unidentified
A_Sordariomycetes (Pezizomycotina)
A_Saccharomycetes (Saccharomycotina)
A_Pezizomycetes (Pezizomycotina)
A_Orbiliomycetes (Pezizomycotina)
A_Leotiomycetes (Pezizomycotina)
A_Eurotiomycetes (Pezizomycotina)
A_Dothideomycetes (Pezizomycotina)
