# Supplementary material for: Effects of the Denitrification Inhibitor “Procyanidins” on the Diversity, Interactions, and Potential Functions of Rhizosphere-Associated Microbiome
Source: Microorganisms. 2021 Jun 29;9(7):1406. doi: 10.3390/microorganisms9071406 (PMC8306639; doi:10.3390/microorganisms9071406)
Supplement: Supplementary file 1 [file microorganisms-09-01406-s001.zip › microorganisms-1269648-tex.zip!/Definitions/microorganisms-1269648-g004.pdf]

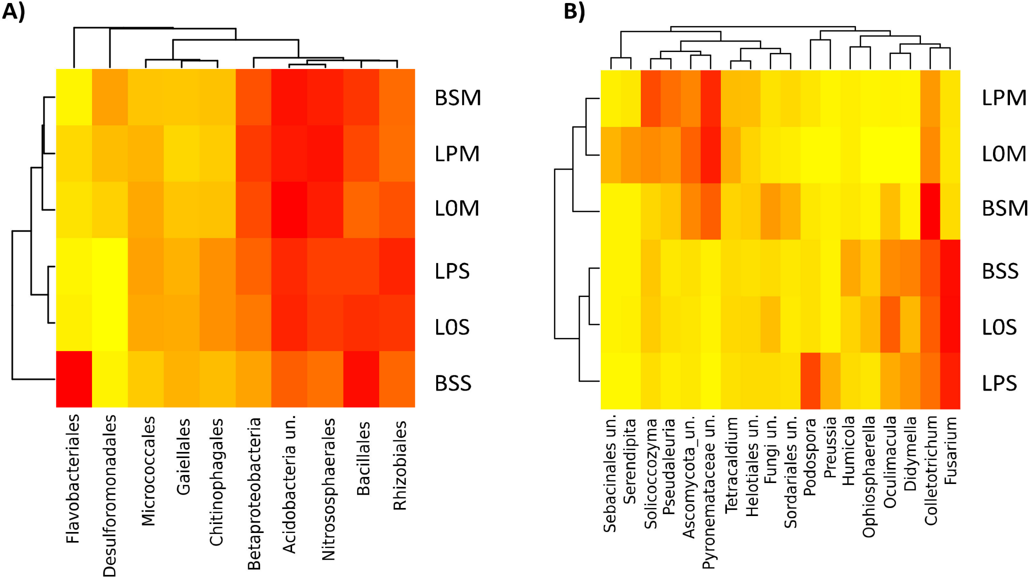
A)
BSM
LPM
L0M
LPS
L0S
BSS
Flavobacteriales
Desulforomonadales
Micrococcales
Gaiellales
Chitinophagales
Betaproteobacteria
Acidobacteria un.
Nitrososphaerales
Bacillales
Rhizobiales
B)
LPM
L0M
BSM
BSS
L0S
LPS
Sebacinales un.
Serendipita
Solicoccozyma
Pseudaleuria
Ascomycota_un.
Pyronemataceae un.
Tetracladium
Helotiales un.
Fungi un.
Sordariales un.
Podospora
Preussia
Humicola
Ophiosphaerella
Oculimacula
Didymella
Colletotrichum
Fusarium
