# Supplementary material for: Effects of the Denitrification Inhibitor “Procyanidins” on the Diversity, Interactions, and Potential Functions of Rhizosphere-Associated Microbiome
Source: Microorganisms. 2021 Jun 29;9(7):1406. doi: 10.3390/microorganisms9071406 (PMC8306639; doi:10.3390/microorganisms9071406)
Supplement: Supplementary file 1 [file microorganisms-09-01406-s001.zip › microorganisms-1269648-tex.zip!/Definitions/microorganisms-1269648-g005.pdf]

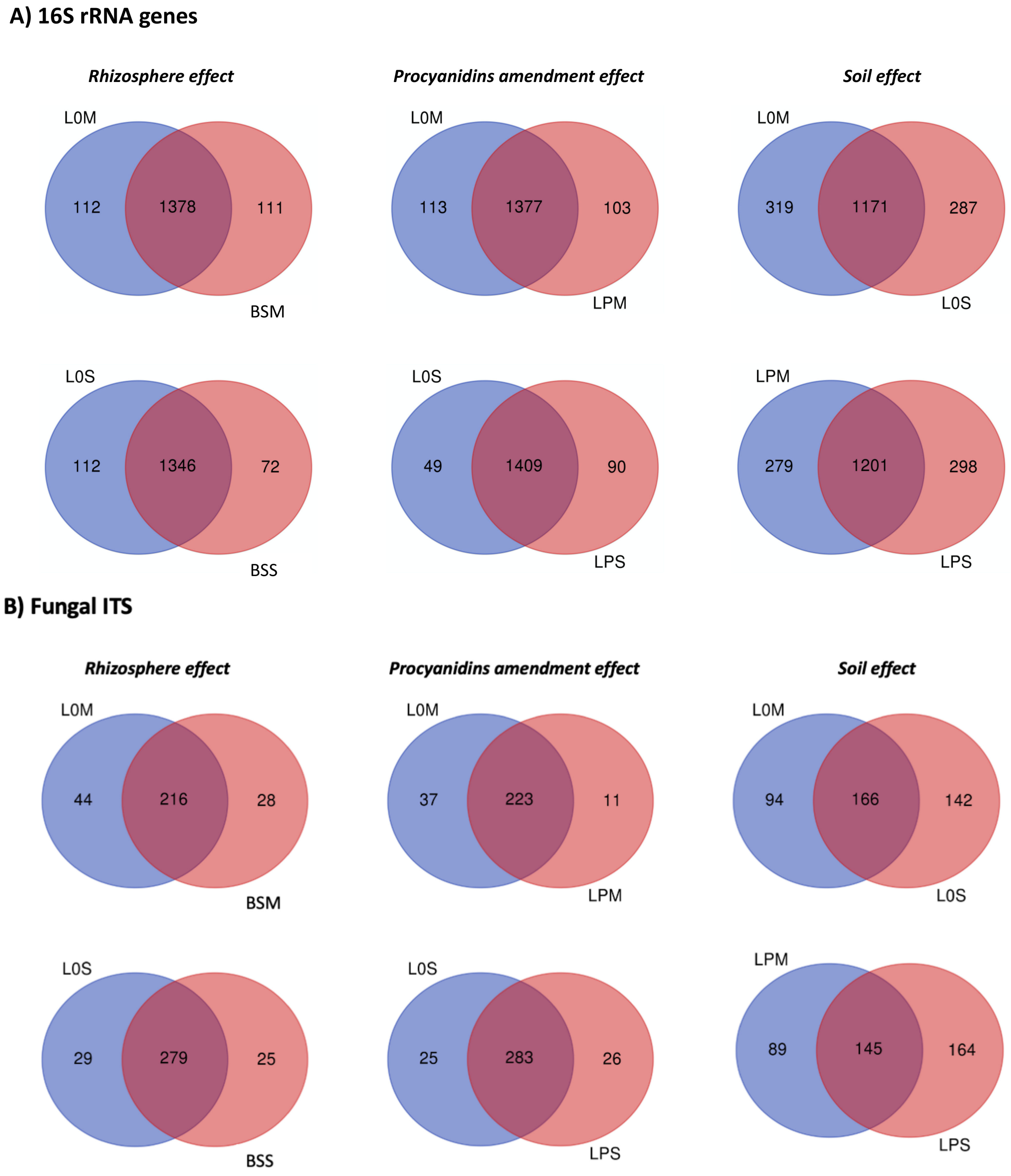
A) 16S rRNA genes
Rhizosphere effect
L0M
112
1378
111
BSM
L0S
112
1346
72
BSS
Procyanidins amendment effect
L0M
113
1377
103
LPM
L0S
49
1409
90
LPS
Soil effect
L0M
319
1171
287
L0S
LPM
279
1201
298
LPS
B) Fungal ITS
Rhizosphere effect
L0M
44
216
28
BSM
L0S
29
279
25
BSS
Procyanidins amendment effect
L0M
37
223
11
LPM
L0S
25
283
26
LPS
Soil effect
L0M
94
166
142
L0S
LPM
89
145
164
LPS
