# Supplementary material for: Effects of the Denitrification Inhibitor “Procyanidins” on the Diversity, Interactions, and Potential Functions of Rhizosphere-Associated Microbiome
Source: Microorganisms. 2021 Jun 29;9(7):1406. doi: 10.3390/microorganisms9071406 (PMC8306639; doi:10.3390/microorganisms9071406)
Supplement: Supplementary file 1 [file microorganisms-09-01406-s001.zip › microorganisms-1269648-tex.zip!/Definitions/microorganisms-1269648-g006.pdf]

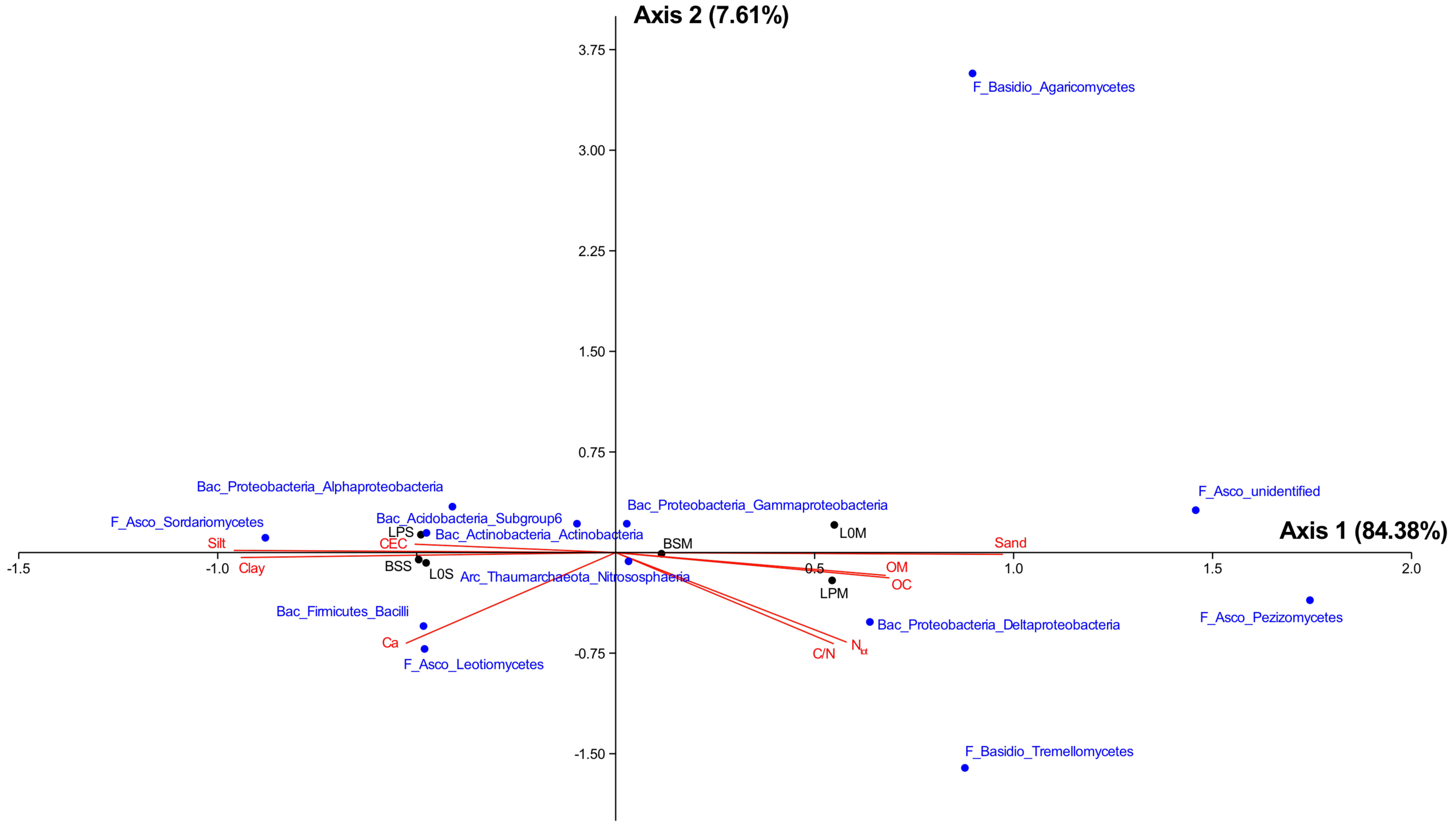
Axis 2 (7.61%)
Axis 1 (84.38%)
3.75
3.00
2.25
1.50
0.75
-0.75
-1.50
-1.5
-1.0
0.5
1.0
1.5
2.0
F_Basidio_Agaricomycetes
Bac_Proteobacteria_Alphaproteobacteria
F_Asco_Sordariomycetes
Bac_Acidobacteria_Subgroup6
Bac_Actinobacteria_Actinobacteria
Bac_Proteobacteria_Gammaproteobacteria
F_Asco_unidentified
Arc_Thaumarchaeota_Nitrososphaeria
Bac_Firmicutes_Bacilli
F_Asco_Leotiomycetes
Bac_Proteobacteria_Deltaproteobacteria
F_Asco_Pezizomycetes
F_Basidio_Tremellomycetes
LPS
BSS
L0S
BSM
L0M
LPM
Silt
Clay
CEC
Ca
Sand
OM
OC
C/N
N_{tot}
