# Supplementary material for: Effects of the Denitrification Inhibitor “Procyanidins” on the Diversity, Interactions, and Potential Functions of Rhizosphere-Associated Microbiome
Source: Microorganisms. 2021 Jun 29;9(7):1406. doi: 10.3390/microorganisms9071406 (PMC8306639; doi:10.3390/microorganisms9071406)
Supplement: Supplementary file 1 [file microorganisms-09-01406-s001.zip › microorganisms-1269648-tex.zip!/Definitions/microorganisms-1269648-g007.pdf]

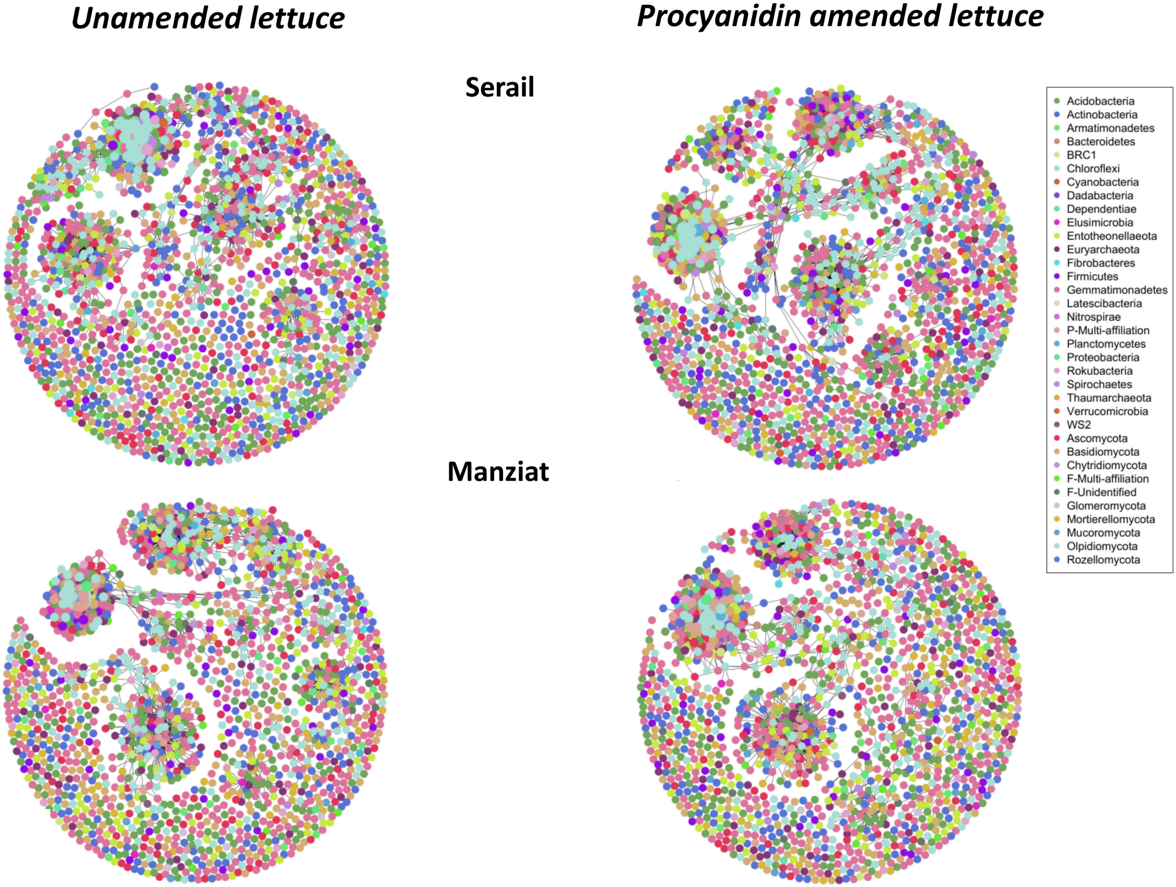
Unamended lettuce
Procyanidin amended lettuce
Serail
Manziat
Acidobacteria
Actinobacteria
Armatimonadetes
Bacteroidetes
BRC1
Chloroflexi
Cyanobacteria
Dadabacteria
Dependentiae
Elusimicrobia
Entotheonellaeota
Euryarchaeota
Fibrobacteres
Firmicutes
Gemmatimonadetes
Latescibacteria
Nitrospirae
P-Multi-affiliation
Planctomycetes
Proteobacteria
Rokubacteria
Spirochaetes
Thaumarchaeota
Verrucomicrobia
WS2
Ascomycota
Basidiomycota
Chytridiomycota
F-Multi-affiliation
F-Unidentified
Glomeromycota
Mortierellomycota
Mucoromycota
Olpidiomycota
Rozellomycota
